# Evaluating the anti-inflammatory and antioxidant efficacy of complementary and alternative medicines (CAM) used for management of inflammatory bowel disease: a comprehensive review

**DOI:** 10.1080/13510002.2025.2471737

**Published:** 2025-03-08

**Authors:** Sia Shin, Siqi Chen, Kangzhe Xie, Suehad Abou Duhun, Tamara Ortiz-Cerda

**Affiliations:** a Sydney Medical School, Faculty of Medicine and Health, The University of Sydney, Sydney, Australia; b School of Medical Sciences, Faculty of Medicine and Health, The University of Sydney, Sydney, Australia; c Departamento de Citología e Histología Normal y Patológica, Facultad de medicina, Universidad de Sevilla, Seville, Spain

**Keywords:** Inflammatory bowel disease, polyphenols, polysaccharides, short-chain fatty acids, probiotics, microbiota, antioxidants, anti-inflammatory

## Abstract

Inflammatory bowel disease (IBD) is a chronic autoimmune condition whose pathogenesis has not been fully elucidated, and current treatments are not definitive and often carry several side effects. The Complementary and Alternative Medicine (CAM) offers a new approach to conventional medicine. However, their clinical application and mechanisms remain limited.

**Objective:** The aim of this review is to evaluate the anti-inflammatory, impact on microbiota and antioxidant efficacy of currently available CAM for IBD.

**Methods:** The literature collection was obtained from Google Scholar, MEDLINE, PubMed and Web of Science (WOS). Studies in both human and animal models, published in English language between 2018 and 2024, were selected. Sixty-seven studies were included in the current review after inclusion and exclusion screening processes.

**Results:** Mostly, studies showed significant anti-inflammatory, gut microbiota restoring, antioxidant effects of polyphenols, polysaccharides, emodin, short-chain fatty acids (SCFA; including butyrate, propionate and acetate), and probiotics although some contrasting results were noted. Current evidence shows that polyphenols exhibit the most consistent result in alleviating IBD pathophysiology, primarily due to their significant SCFA-elevating effect.

**Discussion:** Future studies may focus on human studies, narrowing down on individual factors which may change natural product’s metabolism. Further research studies are also essential to obtain therapeutic recommendations.

## Introduction

Inflammatory bowel disease (IBD) is a chronic and autoimmune condition which consists of Crohn’s Disease (CD) and Ulcerative Colitis (UC) representing the two main subtypes of clinical IBD. Indeterminate Colitis (IC) represents a third subtype of IBD, and this latter diagnosis is made when it is not possible to distinguish between UC and CD. The prevalence of IBD has been estimated in 653 per 100,000 patients, with the number of UC (334 per 100,000) being slightly higher than CD (306 per 100,000) [1]. Epidemiologic studies show that the prevalence of IBD is higher in Europe, North America, and Oceania. However, IBD has evolved into a global disease, with a rising incidence also documented in developing countries in Asia, Africa, and South America [2].

Overall, these subtypes of IBD often present with similar symptoms including abdominal pain, diarrhea, abdominal distension, hematochezia, tenesmus, and an extensive list of extraintestinal manifestations including gastrointestinal, mucocutaneous, musculoskeletal, ocular, pulmonary, vascular system, and generalized fatigue [3], significantly reducing an individual’s quality of life [4]. Contemporary biomarkers include C-reactive protein (CRP), fecal calprotectin (FCP), and lactoferrin, which are all routinely used for the detection of inflammatory activity [5]. However, given the nonspecific symptoms of CD and UC, a diffinitive diagnosis requires confirmation by endoscopy, radiology, and histological analysis of the intestinal tract.

There is currently no cure, and the majority of contemporary therapies focus on symptoms management followed by maintenance of disease remission. Induction and maintenance therapy through the prescription of corticosteroids, 5-aminosalicylates (5-ASA), thiopurines, and biologic therapy trigger adverse effects and have significant impact on patient wellbeing [6]. A perspective population-based cohort study of a total of 330 patients demonstrated that almost 5% of patients with UC required surgery as primary intervention while the corresponding proportion for CD increased markedly to 21.4%. Additionally, the frequency of early postoperative clinical complications remained high in patients for both colon pathologies reaching 31% for UC and 36% for CD [6].

Currently, the pathogenic mechanism of IBD remains unclear [7]; however, factors including a combination of genetic, changes in gut microbiota, and environmental factors (such as diet and lifestyle), as well as individual variability have been well described, ultimately leading to a high reactive oxygen species (ROS) level and activity and an exacerbated colonic immune response in the gut [8].

The upregulation of both innate and adaptive immune responses contributes to colon inflammation and implicates in further tissue damage in patient with UC and CD. Intestinal innate system is made up of neutrophils, monocytes, macrophages, dendritic cells (DCs), and innate lymphoid and natural killer (NK) cells, characterized by their capacity to produce a rapid and nonspecific reaction as a first-line response [9] through the expression of pattern recognition receptors (PRR), such as toll-like receptors (TLR) to identify the molecular patterns of different microorganisms [10]. Moreover, innate cells, specially DCs are responsible to antigen presenting which are key to T-cell activation and the induction of adaptive immune response and in the developing of IBD [11]. Macrophages are also involved in the pathophysiological feature of IBD. For instance, a human study showed that inducible nitric oxide synthase (iNOS), a free radical producing enzyme and a marker of M1 macrophage inflammatory response, was primarily found in active UC, implying that in addition to inflammatory responses, elevated oxidative stress also involved in active state of the disease [12].

Additionally, Schroder et al. [13] have demonstrated a significant increase in inflammatory markers such as neutrophil elastase (NE) and myeloperoxidase (MPO)-indicative of neutrophil extracellular trap (NET) formation in specimens affected by CD compared to control. Similarly, other researchers have shown an overexpression of NET-associated proteins in inflamed colon of UC patients as compared to CD patients and normal control, where patients diagnosed with UC showed a higher capacity of neutrophils to produce NETs upon tumor necrosis factor (TNF)-α stimulation, which is reduced in patients receiving successful treatment with anti-TNF-α [14]. On the other hand, IBD is also characterized by an increase in colonic oxidative stress [15] that can manifest as oxidative damage to a range of biomolecules. In support of this notion, plasma levels of free thiols, a robust biomarker of systemic reduction–oxidation (redox) status, decrease significantly in CD when compared to healthy subjects [16].

Chronicity, unpredictable course of the disease, lack of definitive treatment, and several side effects from current treatment of IBD generate a great interest to study a new therapy with less side effect and higher treatment’s adherence. From this standpoint, Complementary and Alternative Medicine (CAM), a treatment approach that often utilizes natural compounds for pharmaceutical purposes, showcasing a promising complementary option to the conventional medicine, which allows reduced dosage of drugs, frequency or to maintain the remission phase. Have been widely reported the use of bioactive natural compound from plants for pharmaceutical propose. Precedents include aspirin, which is salicylic acid first used reporting back 4000 years by the Sumerians, who obtained it from Willow tree bark [17]. Digoxin, derived from *Digitalis lanata* plant for addressing cardiac issues [18], sterols, their derivative compounds, and plant stanol (phytosterols/phytostanols) for managing hypercholesterolemia [19] are other examples of natural compounds with current clinical use. However, there is yet to be a conventional plant derivative treatment in the field of IBD. Plants are commonly used by IBD patients to alleviate symptoms and most of evidence showed them only as complementary and alternative medicine.

During the coronavirus disease 2019 (COVID) pandemic, an online survey study of IBD patients revealed that 5% of responders ceased or reduced dose of prescribed medications, 13% started supplements such as vitamin D, vitamin C, and other herbal supplements. 43% of responders used CAM and 34% used CAM frequently. Interesting, 59% of CAM users were satisfied and reported it to ‘work well’ or ‘work very well’ and their use was significantly associated with low medication adherence scores and major concern, higher perceived harm and lower score in ‘necessity’ from IBD medications [20]; however, any impact of work from home environment was not reviewed. Additionally, a recent study revealed that the frequency of herbal therapy use, combined with exercise, physical therapy, modified diet, as alternatives to contemporary drug treatments has increased in IBD patients from 2002 to 2019 [21]. Specifically, this study reported that patients experiencing resistance to contemporary treatment options, higher disease activity, or dealing with persistent and severe side effect from standard medications, corticosteroid or use of biologics, and lower quality of life were more likely to seek benefit from CAM [21].

Accordingly, studies examining the therapeutic effects of these plant derivatives in animals provide a versatile preclinical platform for evaluating treatment efficacy and elucidating mechanisms of actions. Experimental model of IBD using chemical stimulators such as dextran sodium sulfate (DSS) or di/trinitrobenzene sulfonic acid (DNBS/TNBS) is frequently studied as a preclinical experimental model, as they manifest clinical and histopathological changes such as irregular stool consistency or diarrhea, bloody stool, and mucosal damage, which are typically observed in IBD patients [22,23].

A complete holistic explanation which enables connection between IBD and potential therapeutic effect of CAM has not been established [24]. This is likely due to the complex nature of herbal mixtures that are available in market, where they are derived from multiple plants and each plant also has countless derivatives individually. Large amount of promising nutraceuticals and natural compounds have been reported for IBD treatment although rigorous evaluation of the benefits is often lacking. These nutraceuticals can be categorized broadly into the following five classes:

- Polyphenols are a class of compounds consisting of one or more phenyl rings combined with one or more hydroxyl moieties and are commonly found in plant products recommended to alleviate gastrointestinal-related discomfort [25] with accumulative evidence supporting their positive effect on intestinal inflammation [26] gut microbiota [27], and redox imbalance that are linked to altered cellular function [28].
- Polysaccharides are common natural macromolecules consisting of covalently linked monosaccharides (generally ≥10 monomer units) that form different polymeric structures [29] with documented biological activity of antitumor, antioxidant, and moisturizing activities, immune protein regulation, improving dendritic cell activity and cytokine release to potentially protect the colon [30].
- The anthraquinone emodin (1, 3, 8-trihidroxy-6-methylanthraquinone) has gained particular interest on experimental models of IBD as recent studies have identified multiple biological actions for emodin with beneficial activities including anti-inflammation and gut-immunity symbiosis, which are all relevant pathophysiological actions central for disease progression in IBD pathogenesis [31,32].
- Short-chain fatty acids (SCFA) represent a series of metabolites produced by gut microbiota commonly categorized by butyric acid, propionic acid and acetic acid, and has been widely recognized crucial to immune homeostasis [33]. Dietary fiber supplements are often incorporated to increase SCFA production, but types of dietary fiber and subsequent types of SCFA produced may lead to different effects on the microbial composition, diversity, and the immune system [34].
- Probiotics may be an essential therapeutic agent which can not only be used as a single agent but to aid conventional therapy as well, as probiotics with nanoenzyme coating therapy led to significant improvement in weight loss, apoptosis, mucin (MUC)-2 level, tight junction proteins, and Disease Activity Index (DAI) [35] and Mesalamine loaded with probiotics showed significant restoration of weight, fecal consistency, fecal bleeding [36].

The aim of this comprehensive review is to evaluate the anti-inflammatory and antioxidant efficacy of currently available CAM for IBD, as well as their role on gut microbiota, and to highlight the underlying molecular and cellular mechanisms of these treatments.

## Methods

### Keyword and search strategy

This review utilized the Population, Intervention, Control, Outcome (PICO) search strategy to examine the anti-inflammatory and antioxidant effect of CAM in IBD. The keywords of ‘IBD patients’, ‘IBD model’, ‘Ulcerative Colitis’, ‘Crohn Disease’, ‘DSS model’, ‘TNBS model’, ‘DNBS model’ for **Population** and ‘polyphenol’, ‘polysaccharide’, ‘anthraquinone’ alternatively ‘emodin’, ‘SCFA’, ‘probiotics’, ‘Complementary and Alternative medicine’, ‘Herbal medicine’, and ‘Plant medicine’ for **Intervention**. Additionally, the keywords of ‘control’, ‘placebo’, ‘IBD therapy’ alternatively ‘mesalazine’ ‘5-ASA’ and ‘corticosteroids’ were used for **Control** strategy and the **Outcome** strategy includes the keywords of ‘anti-inflammation’, ‘gut microbiota’, and ‘anti-oxidation’.

### Article collection and study inclusion and exclusion criteria

Published literature containing the keywords described above was collected from Google Scholar, MEDLINE, PubMed, and WOS and collated into a single file. The collected articles were screened against the following inclusion and exclusion criteria. All processes involving data collection (keyword and search strategy, article collection, study inclusion and exclusion and data extraction) were performed independently by two researches (S.S and T.O.C). Discrepancies in the processes described above were resolved through consultation and discussion with three other researchers (S.C, K.X, and S.A.D).

Studies included based on the collected articles were screened against the following inclusion and exclusion criteria: (1) must involve animal models or human studies; (2) must focus on the gastrointestinal tract affections; (3) must examine the effect of herbal ingredients or natural product derivatives (polyphenols, polysaccharides, anthraquinone [emodin], SCFA, probiotics) on the pathophysiology of IBD, specifically regarding anti-inflammatory properties, antioxidant effects, and gut microbiota dysbiosis. All studies were filtered according to relevancy and date of publication, only including publication within the last 5 years. The studies included are a combination of preclinical animal experiment, randomized control trials, longitudinal cohort study, and observational studies. Studies that did not fall into the aforementioned criteria were excluded from the current review.

During the screening process, studies that were published in English language that clearly established sample size, controls, and statistical analyses were included in this current review. Collected studies that did not satisfy the above-mentioned criteria were excluded and discarded from the current review. Journal impact factor or other journal metric were not one primary consideration for the inclusion and exclusion criteria.

### Data extraction

The current review extracted the following information from the selected articles: experimental method, participants, CAM intervention, and CAM mode of action. Additionally, the active ingredients of the CAM intervention and changes in bioactivities, gut microbiota, and antioxidant capacity were also extracted from the selected articles.

## Results

**Figure 1.**
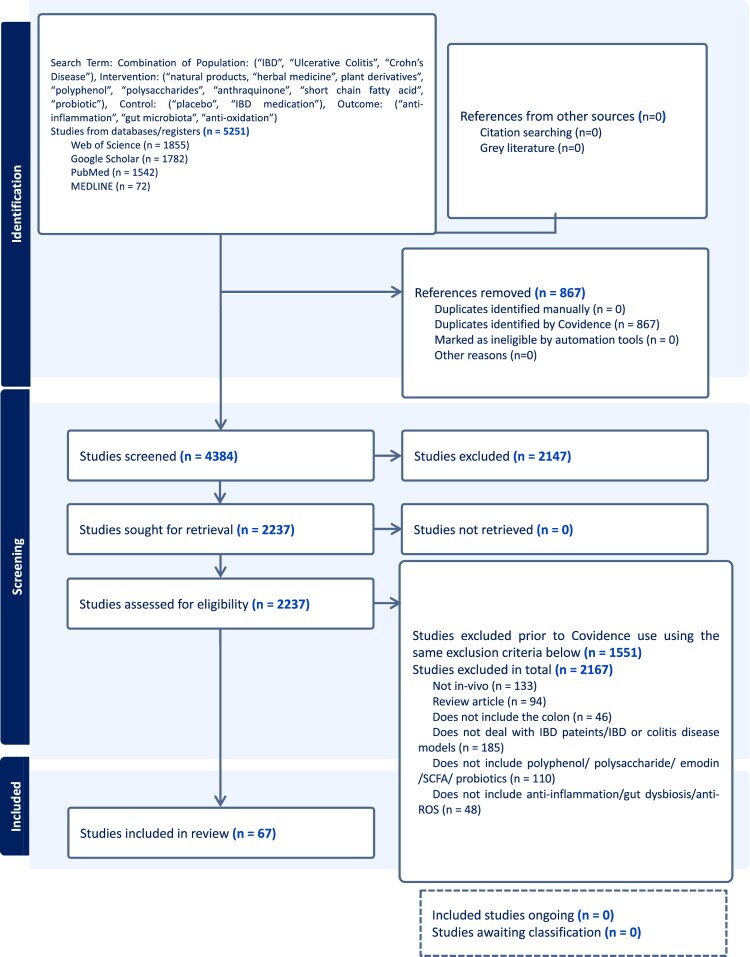
Flow diagram describing the screening strategy to identify the key references used in this systematic review. WOS, google scholar, PubMed, and MEDLINE were used to select all *in vivo* and human studies published in the last 5 years on CAM and IBD. 67 studies were eligible for critical in this review.

The database search began in January 2023 and concluded in August 2024. A total of 5251 articles were obtained. The study inclusion and exclusion screening process was highlighted in the article-screening flow diagram, where a total of 67 studies were included in the current review (Figure 1). The study characteristics were summarized in Table 1.

**Table 1.** Characteristics of screened studies with citations in the Far Left Column.^1^

Ref.	Method	Participants	Intervention	Mode of action
[26]	Preclinical *in vivo* animal experiment	*n* = 32 male ICR mice; 8/group Ethanol-induced inflammation	‘ZAVE’ rich in polyphenols (200 or 800 mg/kg/day), supplemented for 3 weeks	Anti-inflammation, Restoration of gut microbiota
[28]	Preclinical *in vivo* animal experiment	*n* = 48 male C57BL6 mice; 8/group DSS-induced colitis	Apple peel (DAPP) rich in polyphenols (200 or 400 mg/kg/day), for 10 days before and 10 days after induction	Anti-inflammation, anti-oxidation
[37]	Preclinical *in vivo* animal experiment	*n* = 15 male BALB/c mice; 5/group DSS-induced colitis	Resveratrol (100 mg/kg/ day), supplemented for 10 days	Anti-inflammation, restoration of gut microbiota
[39]	Preclinical *in vivo* animal experiment	*n* = 24 male BALB/c mice; 6/group TNBS-induced CD	Polyphenolic maqui extract (50 mg/kg/day) as a preventive (7 days prior TNBS) and therapeutic (4 days after TNBS) administration	Anti-inflammation
[40]	Preclinical *in vivo* animal experiment	*n* = 24 Female C57BL6 mice; 8/group. DSS-induced colitis	EGCG from green tea (50 mg/kg body) supplemented for 3 days	Anti-inflammation, restoration of gut microbiota, anti-oxidation
[49]	Preclinical *in vivo* Animal experiment	*n* = 32 male C57BL/6 mice; 8/group DSS-induced colitis macrophage/ neutrophil depletion	Phenolic acid (50mg/kg) 3 times per time of administration, once in 3 days	Anti-inflammation, restoration of gut microbiota, anti-oxidation
[51]	Randomized Control Trial, Crossover	*n* = 51 Intestinal permeability (↑ zonulin serum level) patients	Diet rich in polyphenols (1391 mg /day), supplemented for 8 weeks	Anti-inflammation, restoration of gut microbiota
[52]	Longitudinal cohort on INCLD Health	*n* = 96 Healthy adults	Culinary herb rich in polyphenols (30,000 PPM, >50,000 PPM, >30,000 PPM/ATB or >50,000 PPM/ATB), tested at 0, 6, and12 months	Restoration of gut microbiota
[106]	Preclinical *in vivo* animal experiment	*n* = 48 male CD-1 mice; 8/group AOM/DSS for CACC	*Moringa oleifera* leaves (5%, 10%, or 20%), supplemented for 12 weeks	Anti-inflammation, anti-oxidation
[119]	Preclinical *in vivo* animal experiment	*n* = 30 male nude mice; 5/group Xenograft for CC	‘SDEA’ flavonoid (100, 200, or 300 mg/kg/day), supplemented for 25 days	Anti-inflammation
[163]	Preclinical *in vivo* animal experiment	*n* = 60 male Sprague-Dawley rats; 10/group DSS-induced colitis	SFE flavonoids (50, 100, or 150 mg/kg/day), supplemented for 1 week	Anti-inflammation
[171]	Preclinical *in vivo* animal experiment	*n* = 36 male & female Labrador Retrievers; 12/group IBD	‘GSP’ rich in proanthocyanidine (30 mg/kg), supplemented for 21 days	Anti-inflammation, restoration of gut microbiota
[172]	Preclinical *in vivo* animal experiment	*n* = 48 male C57BL/6 mice; 8/group DSS-induced colitis	BI extract from Ginkgo biloba (2.5, 5, or 10 mg/kg/day), supplemented for 4–10 days	Anti-inflammation, restoration of gut microbiota
[58]	Preclinical *in vivo* animal experiment	*n* = 55 female C57BL/6J mice; 7–8/group DSS-induced colitis	‘HAPS3a’ and ‘APD3a’ polysaccharides (200 mg/kg), days 2–5 over a total of 5 days	Anti-inflammation, restoration of gut microbiota
[59]	Preclinical *in vivo* animal experiment	*n* = n.i male C57BL/6 mice; n.i/ group DSS-induced colitis	Noni (L.) polysaccharide (10 mg/kg/day), supplemented for 11 days	Anti-inflammation
[60]	Preclinical *in vivo* animal experiment	*n* = 48 male BALB/c mice; 8/group DSS-induced colitis	‘EC’ polysaccharide (0.35, 0.70, or 1.75g/kg/day), supplemented for 7 days	Anti-inflammation
[61]	Preclinical *in vivo* animal experiment	*n* = 60 male BALB/c mice; 12/group DSS-induced colitis	‘DO’ polysaccharide (50, 100, or 200mL/kg/day), supplemented for 7 days	Anti-inflammation
[62]	Preclinical *in vivo* animal experiment	*n* = 40 male & female Sprague Dawley (SD) rats; 10/group Acetic acid-induced colitis	‘HE’ polysaccharide (0.6 or 1.2 g/kg/day) for supplemented 10 days	Anti-inflammation, restoration of gut microbiota, anti-oxidation
[63]	Preclinical *in vivo* animal experiment	*n* = 40 female Swiss mice; 8/group DSS-induced colitis	‘RGal’ polysaccharide (3, 10, or 30 mg/kg/day), supplemented for 7 days	Anti-inflammation
[64]	Preclinical *in vivo* animal experiment	*n* = 15 male C57BL/6j mice; 5/group DSS-induced colitis	‘HAW1-2′ polysaccharide (30 mg/kg/day), supplemented for 3 weeks	Anti-inflammation, restoration of gut microbiota
[65]	Preclinical *in vivo* animal experiment	*n* = 40 male C57BL/6J mice; 8/group DSS-induced colitis	‘AMP’ polysaccharide (10, 20, or 40 mg/kg), 3 days before induction and 7 days post-induction	Anti-inflammation, restoration of gut microbiota
[66]	Preclinical *in vivo* animal experiment	*n* = 36 male Swiss mice; 6/group Acetic acid-induced colitis	Noni-PLS (0.1, 0.3, or 3.0 mg/kg), administered for 30 min before euthanasia	Anti-inflammation, anti-oxidation
[67]	Preclinical *in vivo* animal experiment	*n* = 60 male ICR mice; 20/group AOM/DSS colitis associated colorectal cancer	Apple polysaccharide (10 mg/kg) supplemented for 15 weeks	Anti-inflammation, restoration of gut microbiota
[68]	Preclinical *in vivo* animal experiment	*n* = 50 male BALB/c mice; 10/group Cy-induced immunosuppression	‘TFPS’ heteropolysaccharide (50, 100, or 200 mg/kg/d) supplemented for 10 days	Anti-inflammation, restoration of gut microbiota
[69]	Preclinical *in vivo* animal experiment	*n* = 18 BALB/c mice, Sex n/i; 6/group AOM/DSS colitis associated colorectal cancer	‘EPS1-1’ polysaccharide (150 mg/kg) supplemented for 14 days	Anti-inflammation, restoration of gut microbiota
[70]	Preclinical *in vivo* animal experiment	*n* = 18 male Sprague-Dawley rats; 6/group TNBS-induced colitis	Polysaccharides (300 mg/kg/day), supplemented for 16 days	Anti-inflammation, restoration of gut microbiota, Anti-oxidation
[122]	Preclinical *in vivo* animal experiment	*n* = 24 Male & Female rats; Strain n/i;6/group Healthy rats	‘AOS’ oligosaccharide (200 mg/kg), daily, supplemented orally for 28 days	Anti-inflammation, restoration of gut microbiota
[80]	Preclinical *in vivo* animal experiment	*n* = 80 male & female Kunming mice; 10/group *E. coli* -induced diarrhea	Emodin (8.75, 17.5, or 35 mg/kg) for 6 h before euthanasia	Anti-inflammation, restoration of gut microbiota
[81]	Preclinical *in vivo* animal experiment, *in vitro*	*n* = 150 male BALB/c mice; 30/group CLP-induced sepsis	Emodin (10, 20, or 40 mg/kg), supplemented for 7 days	Anti-inflammation
[82]	Preclinical *in vivo* animal experiment, *in vitro*	*n* = 49 male BALB/c mice; 7/group DSS-induced colitis	Emodin/PSM NPs (5 or 20 mg/kg/d), supplemented for 5 days	Anti-inflammation
[83]	Preclinical *in vivo* animal experiment, *in vitro*	*n* = 48 male BALB/c mice; 8/group CLP-induced sepsis	Emodin (20, 40, or 80 mg/kg/day), supplemented for 5 days	Anti-oxidation
[89]	Human observational study	*n* = 28 UC patients	Butyrate (0–1.6 mM) on blood and intestinal T cells from patients	Anti-inflammation
[173]	Human observational study; Preclinical *in vivo* animal experiment	*n* = 187 IBD patients (CD and UC); *n* = 48 male C57BL/6 mice, 12/group DSS-induced colitis	Butyrate (200 mM), supplemented for 10 days in animal model	Anti-inflammation, anti-oxidation
[12]	Human observational study; Preclinical *in vivo* animal experiment	*n* = 28, UC patients (active and inactive); *n* = 24 male BALB/c mice; 6/group DSS-induced colitis	Butyrate (20 mg/kg), supplemented for 12 days in animal model	Anti-inflammation, restoration of gut microbiota, anti-oxidation
[109]	Human observational study; Preclinical *in vivo* animal experiment	*n* = 112; IBD patients (CD and UC); *n* = 20–40 male C57BL/6J mice; 7–10/group, DSS-induced colitis	‘BHB’ ketone (15 mg/25g), single dose	Anti-inflammation, restoration of gut microbiota
[107]	Preclinical *in vivo* animal experiment	*n* = 44 male C57BL/6 mice; 8–12/group DSS-induced colitis	Butyrate (NaB, 0.1 M or 500 g/kg/day), supplemented 12 days before induction and 10 days post-induction	Anti-inflammation, anti-oxidation
[113]	Preclinical *in vivo* animal experiment	*n* = 24 male C57BL/6 mice; 4/group Streptomycin or *E. coli* strains (LF82 or LF82*lux*)-induced infection	Propionic acid (20 mM), 3 days before infection and 21 days after infection	Restoration of gut microbiota
[112]	Preclinical *in vivo* animal experiment	*n* = 40 male Sprague-Dawley rats; 8/group Health rodent	Cured chicken or beef product diet supplemented for 3 weeks	Anti-inflammation, restoration of gut microbiota, anti-oxidation
[116]	Preclinical *in vivo* animal experiment	*n* = 20 male BALB/c mice; 3–5/group Oxazolone-induced UC	‘DCA’ (100 mg/kg for 3 days), 1 h before induction and 3 days after induction	Anti-inflammation, anti-oxidation
[117]	Preclinical *in vivo* animal experiment	*n* = 10 male C57BL/6 mice; 5/group DSS-induced colitis	Acetate enema (10 mM), provided daily for 7 days	Anti-inflammation
[120]	Preclinical *in vivo* animal experiment	*n* = 20 male C57BL/6J mice; 5/group Fiber-deficient and low DSS-induced colitis	Sodium acetate (200 mM/day), supplemented for 7 days	Anti-inflammation
[35]	Preclinical *in vivo* animal experiment	*n* = 15 C57BL/6 mice Sex n/i; 3/group DSS-induced colitis	Pt-Lipid@EcN probiotic, supplemented for 7 days Dose n/i	Anti-inflammation, anti-oxidation
[36]	Preclinical *in vivo* animal experiment	*n* = 30 female and male Wistar rats; 6/group DSS-induced colitis	Mesalamine and ‘F12’ probiotic (23 mg/kg/day), supplemented for 15 days	Anti-inflammation
[96]	Human observational study; Preclinical *in vivo* animal experiment	*n* = 37 UC patients; n = 30 male BALB/c mice; 6/group DSS-induced colitis	Human FMT, supernant mixture and 7 mix probiotics strains: *E. hirae, L. casei, S. salivarius, F. prausnitzii, A. muciniphila, C. butyricum, L. salivarius (*1 × 10^8^ CFU per strain), administered for 7 days	Anti-inflammation, restoration of gut microbiota
[100]	Preclinical *in vivo* animal experiment	*n* = 60 male C57BL/6 mice; 12/group DSS-induced colitis	Butyrate-producing *Veillonella* and *lactobacillus* (1 × 10^9^ CFU mL^−1^ each, 200 μL per day), supplemented for 14 days	Anti-inflammation, restoration of gut microbiota, anti-oxidation
[102]	Preclinical *in vivo* animal experiment	*n* = 25 male BALB/c mice; 5/group	*Lactobacillus plantarum* ZJ31 (400 uL oral administration, 2.5 × 10^9^ CFU mL^–1^), supplemented for 56 days	Anti-inflammation, Restoration of gut microbiota
[108]	Preclinical *in vivo* animal experiment	*n* = 24 male C57BL/6 mice; 8/group DNBS-induced colitis	Butyrate-producing *Faecalibacterium prausnitzii* strain A2-165 probiotic (1 × 10^9^ CFU), for 10 days after first induction and 3 days after second induction	Anti-inflammation
[111]	Preclinical *in vivo* animal experiment	*n* = 30 male Sprague-Dawley rats; 6/group DSS-induced colitis	*Propionibacterium freudenreichii* ‘LPF’(1 × 10^8^ CFU) and ‘SPFC’ (1 mL), supplemented for 22 days	Anti-inflammation
[121]	Preclinical *in vivo* animal experiment	*n* = 40 male C57BL/6J mice; 10/group DNBS-induced colitis	Acetate-producing bacteria *Christensenella minuta* (1 × 10^9^ CFU/mL), supplemented for 2 weeks	Anti-inflammation
[123]	Preclinical *in vivo* animal experiment	*n* = 32 female C57BL/6J mice; 8/group DSS-induced colitis	*Lactobacillus acidophilus* KBL402 and KBL409 probiotics (1 × 10^9^ CFU), supplemented for 8 days	Anti-inflammation, restoration of gut microbiota, anti-oxidation
[139]	Preclinical *in vivo* animal experiment	*n* = 20 male C57BL/6 mice; 3–7/group DSS-induced colitis	*Lactobacillus johnsonii* probiotic (1 × 10^9^ CFU/day), supplemented for 14 days	Anti-inflammation, restoration of gut microbiota
[140]	Preclinical *in vivo* animal experiment	*n* = 60 male BALB/C mice; 12/group DSS-induced colitis	*NKU556-Fe* (0.2 mg mL^–1^ Fe^2+^), supplemented for 6 days	Anti-inflammation, anti-oxidation
[141]	Preclinical *in vivo* animal experiment	*n* = 70 male Sprague Dawley rats; 10/group DSS-induced colitis	*Lactobacillus acidophilus* (1 × 10^8^ CFU) supplemented for 7 days	Anti-inflammation, restoration of gut microbiota, anti-oxidation
[142]	Preclinical *in vivo* animal experiment	*n* = 18 male C57BL/6 mice; 6/group DSS-induced colitis	*Lactobacillus plantarum* CBT LP3 probiotic (1 × 10^8^ CFU/day), supplemented for 7 days	Anti-inflammation, anti-oxidation
[143]	Preclinical *in vivo* animal experiment	*n* = 16 C57BL/6JOlaHsd mice; 4/group DSS-induced colitis	*Lactobacillus salivarius* probiotic (1 × 10^9^ CFU/day), supplemented for 7 days	Anti-inflammation, restoration of gut microbiota, anti-oxidation
[144]	Preclinical *in -vivo* animal experiment	*n* = 20 female C57BL/6 mice; 5/group DSS-induced colitis	‘Lac@HDP’ probiotic (1 × 10^9^ CFU), supplemented 1 d	Anti-inflammation, restoration of gut microbiota
[146]	Preclinical *in vivo* animal experiment	*n* = 25 female C57BL/6 mice; 5/group DSS-induced colitis	*Porphyromonas gingivalis* and *Lactobacillus rhamnosus* GG probiotics (50 μg mL^−1^), supplemented for 8 days	Anti-inflammation
[148]	Preclinical *in vivo* animal experiment	*n* = 60 BALB/c mice; Sex n/i; 12/group DSS-induced colitis	*Lactobacillus plantarum strains* (1 × 10^9^ or 1 × 10^10^ CFU/mL/day), supplemented for 28 days	Anti-inflammation, restoration of gut microbiota, anti-oxidation
[149]	Preclinical *in vivo* animal experiment	*n* = 32 female C57BL/6J mice; 8/group DSS-induced colitis	*Lactobacillus casei* LH23 probiotic (1 × 10^8^ CFU/day), supplemented for 7 days	Anti-inflammation, anti-oxidation
[150]	Preclinical *in vivo* animal experiment	*n* = 16 BALB/c Wistar Rattus; 8/group DSS-induced colitis	*Lactobacillus brevis*-derived poly *P* probiotic (5 μg/mice/day), supplemented for 7 days	Anti-inflammation
[151]	Preclinical *in vivo* animal experiment	*n* = 90 male C57BL/6N mice; 10/group DSS-induced colitis	*Bifidobacterium bifidum* (FL-276.1, FL-228.1) *Enterococcus faecalis* (ML329, FN249), *Lactobacillus rhamnosus* (FN518), *Lactobacillus fermentum* (CECT5716) (1 × 10^9^ CFU) probiotics, supplemented for 22 days	Anti-inflammation
[152]	Preclinical *in vivo* animal experiment	*n* = 48 female C57BL/6 mice; 8/group DSS-induced colitis	*Bifidobacterium bifidum* BGN4-SK probiotic (1 × 10^10^ CFU/day), supplemented for 8 days	Anti-inflammation, anti-oxidation
[153]	Preclinical *in vivo* animal experiment	*n* = 40 female C57BL/6J and BALB/c ByJ mice; 5–10/group TNBS-induced colitis	*Bifdobacterium animalis* spp. lactis (Bl 5764) and *Lactobacillus reuteri* (Lr 5454) probiotics (1 × 10^8^ CFU/day), supplemented for 5 days	Anti-inflammation
[155]	Preclinical *in vivo* animal experiment	*n* = 12 male C57BL/6 mice; 6/group DSS-induced colitis	*Pediococcus pentosaceus* probiotic (1 × 10^9^ CFU /day), supplemented for 2 weeks	Anti-inflammation, restoration of gut microbiota, anti-oxidation
[156]	Human observational study; *In vivo* animal experiment	*n* = 12 UC patients; *n* = 10 male C57BL/6 mice; *n* = 3–6/group DSS-induced colitis	*Akkermansia muciniphila* (1 x 10^9^ CFU/day), supplemented for 7 days	Anti-inflammation
[157]	Preclinical *In vivo* animal experiment	*n* = 40 male C57/BL6 mice; 10/group	*Akkermansia muciniphila* 139 and ATCC (2 × 10^8^ CFU/ml/day), supplemented for 56 days	Anti-inflammation, anti-oxidation
[158]	Preclinical *in vivo* animal experiment	*n* = 26 male C57BL/6 mice; 6/group DSS-induced colitis	Lactic acid (0.25 mM) and *Saccharomyces cerevisiae* 39# probiotic (1 × 10^9^ CFU/ml), for 7 days	Anti-inflammation, restoration of gut microbiota, anti-oxidation

**ATB**, antibiotic properties; **APS3a**, non-honey-processed *Astragalus* polysaccharides; **AMP**, *Atractylodes macrocephala* Koidz polysaccharide; **AOM**, azoxymethane; **AOS**, alginate oligosaccharides; **BHB**, ketone body B-hydroxybutyrate; **BI**, Bilobalide; **CD**, Crohn’s disease; **CACC**, colitis-associated colorectal cancer; **CC**, colorectal cancer; **Cy**, cyclophosphamide; **CLP**, cecal ligation puncture; **CFU**, colony-forming units; **DSS**, dextran sulfate sodium; **DAPP**, dried apple peel powder; **DO**, Dendrobium officinaleon; **DNBS**, dinitrobenzene sulfonic acid; **DCA**, dichloroacetate; **EGCG**, epigallocatechin-3-gallate; **EC**, *Eucheuma cottonii*; **EPS1-1**, *Rhizopus nigricans* extracellular polysaccharide; **SFE**, *Sophora flavescens* extract; **FMT**, fecal microbiota transplantation; **F12**, probiotic microparticles; **GSP**, grape seed proanthocyanidin; **HAPS3a**, honey-processed *Astragalus* polysaccharides; **HAW1**, *Crataegis pinnatifida* (Hawthorn); **‘HE’**, *Hericium erinaceus*; **INCLD**, International Cohort on Lifestyle Determinants of Health; **LPF**, live *P. freudenreichii* KCTC 1063; **Lac@HDP**, *Lactobacillus acdiphilus*@Hyaluronic acid grafted with dopaine protected by phenylboric acid; **Noni-PLS**, isolated polysaccharide from *Morinda citrifolia* Linn; **NPs**, nanoparticles; **NaB**, sodium butyrate; **NKU556-Fe**, *Lactobacillus alimentarius* NKU556 iron-enriching from Chinese fermented food; **n/i**, no-information provided; **PPM**, parts per million; **PSM**, poly (DL-lactide-co-glycolide)/Eudragit^Ⓡ^ S100/montmorillonite; **Pt-Lipid@EcN**, liposome-coated *Escherichia coli* Nissle (EcN) 1917; **Poly *P***, long-chain polyphosphate; **RGal**, Rhamnogalacturonan; **SPFC**, supernatant of the *P. freudenreichii* culture; **SDEA**, *Selaginella doederleinii Heiron* etyl acetate; **TNBS**, 2,4,6-Trinitrobenzene sulfonic acid; **TFPS**, *Camellia sinensis* L pectic heteropolysaccharides; **UC**, *Ulcerative colitis*; **ZAVE**, Zhenjiang aromatic vinegar extract.

### Polyphenols and their bioactive metabolites

Natural polyphenols are a class of compounds consisting of one or more phenyl rings combined with one or more hydroxyl moieties. These compounds are commonly found in plant products and are often recommended to alleviate gastrointestinal-related discomfort [25]. Accordingly, polyphenols are increasingly gaining attention as a potential therapeutic agent for IBD, with accumulated evidence supporting their positive effects on intestinal inflammation [26,28], gut microbiota [37,38], and redox imbalances that are linked to altered epithelial cell function [28].

#### Polyphenols as anti-inflammatory agents

Studies with experimental animals consistently show that polyphenols administration significantly increased colon length, and alleviated inflammatory cell infiltration, weight loss, fecal bleeding, improved stool consistency, and colonic crypt depth in the IBD experimental animal model [26,28,39]. These gross observations were accompanied by significant decrease in proinflammatory biomarkers and inhibition of altered mitochondrial morphology in colon epithelia due to inflammation and provided the basis for the mechanism of action for this class of natural products. Importantly, these combined factors may be critical in determining severity of extraintestinal manifestations hence polyphenols may ameliorate these pathological changes to the colon. In addition, significant relative increases in populations of resolving macrophage phenotype (M2) cells and anti-apoptotic protein expression such as Bcl2, and lower level of pro-apoptotic protein expression, plasma inflammatory biomarkers and facilitators such as interleukin (IL)-6, IL-8, IL-1β, iNOS, cyclooxygenase-2 (COX-2), and TNF- α are consistently reported in pre-clinical interventional studies with polyphenols in mice models of UC and CD-like colitis model [28,39,40]. As anti-inflammatory effects generally show a parallel link to the inhibition of oxidative stress in *in-vitro* studies, it may imply that the cumulative data obtained with pre-clinical models may also contribute to anti-ROS activity that leads to decreased oxidative damage [41], which will be further explored in the section below. In summary, the available evidence largely demonstrates that polyphenols protect against intestinal damage and alleviates both colonic signs and serum biomarkers of inflammation, implying its therapeutic potential for IBD.

#### Polyphenols as scavengers of reactive oxygen species (antioxidant capacity)

ROS are products of cell metabolism or environmental factors such as diet and smoking, which through excessive accumulation can induce host tissue damage [42]. Furthermore, cellular production of ROS is tightly related to inflammatory actions which is a crucial part of disease manifestation in IBD. Local production of ROS may disrupt intestinal permeability, damaging cells which form tight junctions along the colon epithelium [43]. For example, the free radical superoxide radical anion is chemically reduced to hydrogen peroxide (H_2_O_2_, a secondary type of ROS), by superoxide dismutases (SOD1/2). This weak two-electron oxidant H_2_O_2_ is then reduced further by catalase and other professional peroxidase enzymes to eliminate the oxidant*.* Indeed, polyphenol-rich dried apple peel powder showed lower levels of H_2_O_2_ coupled with decreased SOD2 expression, commensurate with a decrease in tissue oxidative stress resulting from inhibiting ROS production [28]. It also enhanced antioxidative action by slowing oxidant production through promoting the master antioxidant transcription nuclear factor erythroid 2-related factor 2 (Nrf-2) linked with enhanced heme oxygenase 1 (HO-1 activity) and reduced iNOS levels through polyphenol treatment [28,39], suggesting that these natural antioxidants may also play a role in alleviating nitrosative stress. Similarly, green tea polyphenol was shown to alleviate suppression of total SOD and catalase (CAT), which modulates H_2_O_2_ levels in cells due to inflammation, leading to the suppression of malondialdehyde (MDA), a marker of oxidative lipid damage [40]. Further *in-vitro* studies have shown that polyphenol derived from an original fruit from south of Chile called ‘maqui’ readily attenuates oxidative stress in a dose dependently manner without the evidence of toxicity, which suggests that this same mechanism may apply *in vivo* [41]. Altogether, the available evidence shows that polyphenols consistently alleviated oxidative stress and inflammation in the colonic mucosa.

#### Polyphenols as modulators of the gut microbiota

Commensal gut microbiota is important for the maintenance of physiological homeostasis whereas pathogenic microbiota can lead to gastrointestinal disorders such as IBD [44]. Notably, in patients exposed to antibiotics or with diets containing high fat and/or low fiber intake, a disproportionate growth of pathogenic microbiota can occur [45]. The gut microbiome has an intricate relationship with not only the intestine but also the brain and other organs such as the liver, which may suggest potentially systemic effects of gut microbiota; its effect on extraintestinal manifestations of IBD are termed the gut-health axis [46]. Previous studies have shown that IBD patients display altered gut microbiota composition, abundance, and diversity, which in combination with a loss in functional epithelial junctions exacerbates inflammation through increasing intestinal permeability [47,48]. An individual’s gut microbiome is influenced by interplay with diet and other environmental factors, as well as conventional IBD medications and other prescribed therapeutics, including antibiotics [49,50]. Polyphenols can improve the gut microbiome state and assist in maintenance of a physiological balance in the gut.

Plasma levels of polyphenol metabolites such as theobromine and methyxanthine showed a significant positive correlation with butyrate-producing bacteria strains such as *Butyricicoccus*, *Faecalibacterium*, and *Lactonifactor*. Polyphenols rich diet regulated gut microbiota, increased abundance of *porphyomonadaceae*, and modulated zonulin levels, which affects intestinal permeability [51]. In further support, a negative correlation with polyphenol-rich diet was seen with pathogenic bacterial strains including species from the Proteobacteria phylum or the Bacteroidales order [51]. Similarly, data analysis of the INCLD (International Cohort on Lifestyle Determinants) described that the high frequency of polyphenol-rich culinary herb use shows a positive association with *Firmicutes* and negative association with *Proteobacteria* in a healthy cohort, albeit no significant association with alpha diversity for a given microbiome was identified [52].

Interestingly, *Firmicutes* showed a conflicting result, with a significantly negative correlation with polyphenol-rich aromatic vinegar consumption [26]. Although this polyphenol-rich aromatic vinegar significantly increased Reg3b and Reg3g antimicrobial peptides that are positively correlated with Bacteroidetes, *verrucomicrobiota*, *Akkermansia*, and Lachnospiraceae_NK4A136. Proteobacteria and Deferribacteria were also negatively correlated with polyphenol supplementation and these bacteria are known to be associated with inflammatory proteins and oxidative biomarkers [26].

Additional evidence indicates that oral intake of polyphenol led to an increase in abundance of *Akkermansia* in the gut and similar increases in propionate and several butyrate-producing taxa [40]. Indeed, the production of the short chain fatty acids propionate and butyrate measured in feces was significantly higher in the polyphenol treatment group. Rectal administration was not as effective as oral, implying metabolism along the digestive tract is critical to the physiological benefit of this form of polyphenol. In terms of bacterial taxa, *Akkermansia* had positive correlation with the antioxidant enzyme CAT levels in plasma, total antioxidant capacity (T-AOC) assessed in the colon, as well as levels of the short-chain lipids acetate, and butyrate in feces, and significantly negatively correlated with the cytokine IL-8. On the other hand, increases in the population of *Lactobacillus* in experimental colitis were paralleled by a distinct negative correlation with colonic TNF-α levels [40].

Table 2 summarizes the compiled action of different plant-derived polyphenols on IBD pathophysiology including anti-inflammation, gut microbiota, and ROS. As mentioned above, different studies have described polyphenol’s inflammatory action in terms of clinical severity, immune cells infiltration, inflammatory cytokines in serum and colon, and macroscopic and histoarchitecture signs in colonic tissue. Significant changes in gut microbiota composition and abundance are also described.

**Table 2.** Mechanism of actions of polyphenols in alleviating IBD with reference sources cited in the far-left column.

Ref.	Active ingredient (where identified) and dose	Chemical stimulus	Species	Bioactivity summary	Change in gut microbiota	Proposed antioxidant mechanism
[26]	Zhenjiang aromatic vinegar polyphenolic extract; 200 and 800 mg/kg	2g/kg1stw, 4g/kg 2^nd^w 6g/kg 3–4^th^ w	Male ICR mice, *n=32*; 8/group	↓ IL-6, ↓ TNF-α ↓ IL-1β.	↑ Antimicrobial peptides (Reg3b and Reg3g) ↑ *Akkermansia,lachnospiraceae_NK4A1* ↑ *Bacteroidetes* ↓ *Firmicutes, Bilophila* and *butyricimonas*	-
[28]	Apple peel polyphenols; 200 and 400 mg/kg	DSS (2.5%)	Male C57BL6 mice *n=48*; 8/group	↓ Infiltration immune cells ↓ mt. abnormalities. ↓ TNF-α, COX-2, iNOS.	-	↑ Nrf-2 ↓Ox-mtDNA, H_2_O_2_. ↑ 8-oxoguanine DNA glycosylase
[37]	Resveratrol; 100 mg/kg	DSS (3%)	Male BALB/c mice *n = 15*; 5/group	↓ Macroscopy damage ↑ Occludin, Claudin 1, PCNA, ↑ IL-10 ↓IL-1β, IL-6 and TNF-α	↑ microbiome diversity ↑ *Bacteroidetes* and, *Proteobacteria*. ↓ *Lachnoclostridium, Acinobacter* and *Serratia*	-
[39]	Polyphenolic Maqui extract; 50 mg/kg	TNBS (100 mg/kg)	Male BALB/c mice *n = 24*; 6/group	↓ Histology damage ↑ M2 macrophage ↓ COX-2	-	↑ Nrf-2/HO-1
[40]	EGCG from green tea; 50 mg/kg body	DSS (2.5%)	Female C57BL6 mice; *n* = 24; 8/group	↑ Body weight ↑ Colon length ↓ DAI ↓ Histological score ↓IL-1β, IL-6, IL-8, and TNF-α	↑ *Akkermansia* ↑ *Lachnoclostridium* ↑ *UBA1819* ↑ *Colidextribacter* ↑ Butyrate ↑ Propionate	↓ MPO ↓ MDA ↑ GSH-Px ↑ CAT ↑ SOD
[49]	CGA, FA, CA and EA fenolic acids; 50 mg/kg	DSS (3%) + 4 ABX cocktail.	Male C57BL/6 mice, *n = 32*; 8/group	↓ Clinical and histological score **CGA:** ↓ M1 and Nlrp3 activation. **FA:** ↓ NETs	UroA-derived EA by gut microbiota ↑ barrier function and IL-22	-
[51]	Diet polyphenols (theobromine and methylxanthine); 1391 mg/day	-	Humans, Intestinal permeability *n = 51*	-	↑ *Alistipes onderdonkii, Anaerobutyricum hallii, Faecalibacterium prausnitzii, Lactonifactor* and *Butyricicoccus* genera. ↓ *Bacteroides uniformis* and *Streptococcus agalactiae*.	-
[52]	Culinary herb containing 30,000–90,000 PPM polyphenols and antibiotic effect.	-	Humans, *n = 96*	–	↑ *Firmicutes* in >30,000 and >90,000 PPM ABX; 30,000 and >50,000 PPM polyphenols. ↓ proteobacteria in >30,000 and >50,000 PPM polyphenol; >90,000 PPM antibiotic	-
[106]	*Moringa oleifera* leaves; 5%, 10%, 20% concentration	AOM (10 mg/kg) + DSS (1.5%)	Male CD-1 mice *n = 48*; 8/group	↑ Cecal SCFA (↑acetic, propionic, butyric acid), ↓ Total polyps ↓ Colonic weight/length ratio ↓ IL-6, TNF-α and MCP-1 ↑ IL-10	-	↑ NQO1 activity ↑ GST ↓ MPO and MDA
[119]	Selaginella doederleinii flavonoid extract; 100, 200, or 300 mg/kg	Xenografts HT29 or HCT116 model	Male nude mice, *n = 30*; 5/group	↓ tumor weight ↓ CD34 expression ↑ apoptosis- and autophagy-related proteins. ↓ Ki67	-	-
[163]	*Sophora flavescens* flavonoid extract; 50, 100 or 150 mg/kg	DSS (5%)	Male Sprague-Dawley rats, *n = 60*; 10/group	↑ colon length ↑ body weight ↓ histology damage	-	-
[171]	Grape seed pro-anthocyanidine; 30 mg/kg	Pre-existing mild IBD	Males & females, Labrador Retrievers, *n = 36*; 12/group	↓ serum CRP, HMGB1, TNFα, IL-6, IL-1β, DAO ↑ IL-10	↑ Microbiome diversity ↑ *Ruminococcaceae, Faecalibacterium*, and *Lachnospiraceae_NK4A136*	-
[172]	*Ginkgo biloba*-extracted bilobalide; 2.5, 5, or 10 mg/kg	DSS (2%)	Male C57BL/6 mice *n = 48*; 8/group	↑ Body weight and colon length ↓ Clinical score ↓ Histological score ↓ IL-1, IL-6, TNF-α ↓ AKT/ NF-κB p65 p38 ↓ ERK and JNK ↑ Claudin-3, Occludin, ZO-1	↑ *Firmicute* and *Lactobacillus* ↓ *Bacteroidetes* and Dubosiella	↓ MPO

**ABX**, antibiotic; **AOM**, azoxymethane; **AKT**, protein kinase B; **COX-2**, cyclooxygenase-2; **CGA**, chlorogenic acid; **CA**, caffeic acid; **CRP**, C-reactive protein; **DAO**, diamine oxidase; **DSS**, dextran sodium sulfate; **EA,** ellagic acid; **ERK**, extracellular signal-regulated kinase; **FA**, ferulic acid; **GSH-Px**, glutathione peroxidase; **GST**, glutathione-S-transferase; **H_2_O_2_**, hydrogen peroxide; **HMGB1,** high mobility group protein B; **iNOS**, inducible nitric oxide synthase; **IBD**, inflammatory bowel disease; **JNK**, C-Jun N-terminal kinases; **Mt.**, mitochondria; **MCP-1**, monocyte chemoattractant protein 1; **MPO**, myeloperoxidase; **MDA**, malondialdehyde; **Nrf-2**, nuclear factor erythroid 2-related factor 2; **NQO1**, NADH-quinone reductase; **PPM**, parts per million; **PCNA**, proliferating cell nuclear antigen; **TNF-α**, tumor necrosis factor-alpha; **UroA**, urolithina; **w**, week; **ZO**-**1**, Zonula occludens 1.

### Polysaccharides

Polysaccharides are common natural macromolecules consisting of covalently linked monosaccharides (generally ≥10 monomer units) that form a variety of different polymeric structures [29,53]. The bioactivity of each polysaccharide is dependent on the differing structures within the polymeric compound and their physico-chemical properties, which can be modulated through chemical modification. Documented biological activities of polysaccharides include antitumor, antiviral, antioxidant, and moisturizing activities [29]. In addition, this class of natural product can regulate cellular release of cytokines such as interferon (IFN)-γ and IL-10 also revealing that polysaccharides can modulate dendritic cell activity, leading to an increased the expression of CD80 and CD86 in these immune cells, and enhancing their antigen presentation ability, which may act in concert to potentially protect the colon from pro-inflammatory stimuli [30]. Additionally, chemical modification via carbomethylation enhances water solubility and further improves the antioxidant capacity of polysaccharides [54]. Further studies are necessary to reveal whether the same chemical modification of different polysaccharide types yields consistent improvement in these bioactivities and whether it can be applied to polysaccharide consumed by humans. For the reasons outlined above, both crude and semi-purified polysaccharide extracts have gained recent attention as a potential natural therapeutic agents. Although polysaccharides, such as that isolated from tea, are influenced by factors such as variation across agriculture regions, age of plant, extraction methods, and integrity on storage, the potential for natural polysaccharides to provide useful therapeutic advantage for IBD is now increasingly recognized [55].

Previous studies have described multiple effects of polysaccharides including enhancing macrophage mediation and immune action and promotion of inflammatory signaling, stimulation of T- and B-cell proliferation, and release of cytokines such as IL-2, INF-γ, IL-4, IL-10, granulocyte-macrophage colony-stimulating factor (GM-CSF), and TNF-α while inhibiting regulatory T-cell action, which may seem counterintuitive as a therapy for IBD [56]. However, a different study revealed polysaccharide was able to regulate macrophage action and prevent 4T1 cell apoptosis, implying an additional cell regulatory effect [57]. This bioactivity was noted in addition to the regulated release of cytokines mentioned above, while the regulation of the natural killer cell population was also noted [60]. The study described above also described the effect of polysaccharide on thymus and spleen function, both major organs of the immune system [57]. This has implications on thyroid hormone-regulatory pathways that a key to physiological homeostasis commensurate with a thyroid–microbiome–immunity axes.

#### Polysaccharides as anti-inflammatory agents

Directly related to IBD, polysaccharide administration reduced shortening of colon, ulceration, mucosal edema, and inflammatory biomarker expression, showing significant alleviation of and protection against mucosal damage in preclinical animal models [58–61]. The evidence of colonic ulcer healing was also documented after polysaccharide therapy that decreased neutrophils and monocytes infiltration into the colon wall [62]. Polysaccharide treatment also led to the restoration of collagen and colon wall histoarchitecture [63]. Nonetheless, other studies have been less favorable. For example, polysaccharide extract from Hawthorn berry (*Crataegus pinnatifida*) did not significantly reduce gross macroscopic damage such as colon shortening, albeit this polysaccharide suppressed inflammatory cytokine expression within the colon wall [64].

The suppression of inflammatory cytokines has been supported by multiple reports in the available literature. For example, Wu et al. [58] showed that polysaccharide extract from Astragalus decreased the levels of inflammatory cytokines in colon of mice, reaching levels similar to those determined in the control group, with levels reduced to near background determined in the control group. Furthermore, this anti-inflammatory action was suggested to be a result of the polysaccharide extract promoting the gut production of SCFAs, leading to the reduction of IL-1β [58]. Similar studies with other polysaccharides have also corroborated a decrease in other pro-inflammatory cytokines IL-1β and IL-18, and nucleotide-binding domain, leucine-rich containing family, pyrin domain containing-3 (NLRP3) inflammasome signaling pathway through marked decrease in the protein level of NLRP3, apoptosis-associated speck-like protein containing a caspase recruitment domain (ASC), caspase1, IL-1β, IL-18 [61] and a concomitant increase in anti-inflammatory IL-10 which occurs in a dose-dependent manner [60,61]. Furthermore, another study showed that polysaccharide consumption at intermediate (20 mg/kg) or high dose (40 mg/kg) significantly reduced disease indices upon both macroscopic and histopathological evaluations of the colon in experimental IBD. A parallel decrease in inflammatory cytokine levels in the colon including TNF-α, IL-1β, once again highlighted the dose-dependent anti-inflammatory effects of dietary polysaccharide [65].

This outcome was further reinforced when rodents were treated with a higher dose of 3.0 mg/kg polysaccharide, compared to 0.1 and 0.3 mg/kg, showed marked resistance to chemically stimulated colitis [66]. Here, dietary supplementation with the natural product manifested in significantly lower colonic wet weight compared to the colitis and control (saline) groups, but lower doses of the same polysaccharide did not protect the colon from experimental colitis [66]. Higher colonic ratio of pro-inflammatory/anti-inflammatory cytokines returned to the normal level, implying the suppression of pro-inflammatory factors in the colon. Interestingly, RGal polysaccharide showed the same degree of significant attenuation of disease activity, histopathological damage and improved colon length irrespective of dose at 3, 10, and 30 mg/kg [63]. These contrasting data suggest that if polysaccharide compounds were to be considered as a therapeutic approach for IBD, standardization of effective dosing for specific types of polysaccharide is likely required for optimal individual therapeutic benefit.

Dietary supplementation with polysaccharide has also led to significantly reduced T-cell and macrophage levels in the colon after 15 weeks of diet supplementation, showing similar T-cell level to the corresponding control group, however, other immune cell types (such as dendritic cells and neutrophils) remained unaffected, which is a common histopathological outcome for experimental IBD [67]. Also, polysaccharide administration significantly alleviated immunosuppression by cyclophosphamide, by acting to reverse the suppression of mRNA expression of cytokines including signal transducer and activator of transcription 3 (STAT3), janus kinase 2 (JAK2), iNOS, Claudin 1 and 5, Occludin-1, TNF-α, IL-1β, TLR4, MyD88, p62, p65 leading to dose-dependent elevation of TNF-α, IFN-γ, IL-1β, IL-2, IL-6 in the circulation [68]. This implies polysaccharide being able to not only suppress the inflammatory immune system but upregulate its expression when necessary. This may lead to the possibility of attenuating immunosuppression due to corticosteroids which is commonly used in IBD patients. In a colorectal cancer mice, polysaccharide therapy has been shown to significantly improve mucosal damage, such as crypt depth and villus length comparable to the model group, and to significantly increase acid and mucus-secreting goblet cells [69]. Thus polysaccharide may assist the immune system not by the preventing gross damage of goblet cells alone, but by aiding their function.

Polysaccharide therapy showed significant results in alleviating colonic damage and suppressing inflammatory cytokines in experimental IBD, but whether the effect is dose dependent and the precise mechanism of action leading to attenuated mucosal damage requires further elucidation. Whether polysaccharide regulation of the immune response may be beneficial for patients who are already immunosuppressed also requires further evaluation.

#### Polysaccharides as anti-oxidants

In summary, the available data suggests that polysaccharide therapy displays a significant antioxidation effect. For instance, high dose of polysaccharide (3.0 mg/kg) significantly improved colonic concentration of the low-molecular weight antioxidant glutathione (GSH) and reduced iNOS expression in the colon mucosa. Moreover, all doses (0.1, 0.3, 3.0 mg/kg) reduced levels of neutrophil MPO but only medium (0.3 mg/kg) and high doses (3.0 mg/kg) significantly ameliorated oxidative damage (judged by levels of colonic lipid oxidation; MDA) and total nitrate/nitrite (NO_3_/NO_2_) levels, suggestive for a dose-dependent effect [66]. Similarly, polysaccharide therapy increased total SOD protein and decreased MDA levels in parallel, however, had no significant effects on C4 and IgG levels [62] and showed only moderate decline in MPO levels after polysaccharide therapy [70]. On balance polysaccharide supplementation has a positive role in reducing oxidation and aiding antioxidant effect in the colon as detailed in the tabulated summary of bioactivity below (Table 3).

**Table 3.** Mechanism of actions of polysaccharides in alleviating IBD with reference sources cited in the far-left column.

Ref.	Active Ingredient (where identified) and dose	Chemical stimulus	Animal	Bioactivity summary	Change in gut microbiota	Proposed antioxidant mechanism
[58]	HAPS3a and APS3a Astragalus Polysaccharide; 200 mg/kg	DSS (3%)	Female C57BL/6J mice, *n = 55*; 7–8/groups	↑colon length, ↓ IL-1β	HAPS3a: ↓ F/B ratio, ↑ Acetic acid and propionic acid concentration APS3a: ↑ butyric acid concentration, ↑ *Firmicutes* ↓ *Verrucomicrobia*	-
[59]	Noni (*Morinda citrifolia* L.) Polysaccharide; 10 mg/kg	DSS (2%)	Male C57BL/6 Mice, *n = n/i;* n/i/groups	↓ Clinical score ↓ Histological score ↑ goblet cell ↑ ZO-1 and occludin	-	-
[60]	*Eucheuma cottonii* polysaccharide extract; 0.35, 0.70 and 1.75 g/kg	DSS (2.5%)	6 week old male BALB/c mice, *n=48*; 8/group	↓ Colon length ↓Clinical activity. Restored crypt morphology ↓TNF-α, IL-6, IL-1β ↑ IL-10	-	-
[61]	*Dendrobium officinale*on polysaccharide; 50, 100, or 200 mg/kg	DSS (4%)	Male BalB/c mice, *n = 60;* 12/group	↓ Clinical and disease activity ↓TNF-α, ↓Inflammasome complex NLRP3 and ↓ IL-18. ↓IL-1β and IFN-γ at 200 mg/kg only	-	-
[62]	*Hericium erinaceus mycelium*; 0.6 or 1.2 g/kg	Acetic acid (4%)	Male & Female Sprague Dawleyvrats *n = 40*; 10/group	↓ Mucosal damage, necrosis, ↓ IL-1 and IL-6	Restoration of *Firmicutes, Bacteroidetes, Proteobacteria*, and SCFA.	↑ SOD ↓ MDA
[63]	Rhamnogalacturonan polysaccharide, 3 and 10 or 30 mg/kg	DSS (5%)	Female Swiss mice *n = 40;* 8/group	↓DAI, weight loss, ↑colon length	-	-
[64]	Crataegis pinnatifida polysaccharide; 30 mg/kg	DSS (3%)	Male C57BL/6j mice, *n = 15*; 5/group	↓ IL-1β, IL-6, TNF-α, ↓ IKKA/B (p89), *p*-IkBα, ↓ NK-kB (p65)	↑ *Alistipes, Dubostella*, *Odoribacter*. ↑ SCFA-producing microbiota ↑Bacteroidetes/Firmicutes.	-
[65]	*Atractylodes macrocephala* Koidz. Polysaccharide; 10, 20, or 40 mg/kg	DSS (2.5%)	Male C57BL/6J mice, *n = 40*; 8/group	↓ DAI ↓ Histological score ↓TNF-α and IL-1β	↑ *Lactobacillus* and *Butyricoccus.* ↓ *Actinobacteria, Verrucomicrobia, Bifidobacterium, Tenericutes, Faecalibaculum, Parasutterella, Akkermansia, Erysipelatocostridium, Anaeroplasma* and *Parvibacter.* Partial restoration of microbiota richness & diversity	-
[66]	*Morinda citrifolia* Linn, polysaccharide; 0.1, 0.3, 3.0 mg/kg	Acetic acid (6%)	Male Swiss mice *n = 36*; 6/group	↓ Colonic wet weight ↓ Histological markers ↓COX-2 and iNOS ↓TNF-α and IL-1β	-	↑GSH at 3.0 mg/kg only. ↓MPO and MDA, ↓NO3/NO2 level.
[67]	Apple polysaccharide; 10 mg/kg	DSS (2%)	Male ICR mice, *n = 60*; 20/group	↓ Histological score, ↓T-cell ↓ Macrophage/monocyte	↓ *Firmicutes* ↑ *Bacteroides*	-
[68]	*Camella sinensis* L., Polysaccharide; 50, 100, or 200 mg/kg	Cy (80mg/kg)	Male BALB/c mice, *n = 50*; 10/group	↑ Body weight ↑ TNF-α, INF-y, IL-6, IL-1β, and IL-2 ↑ STAT3,↑ JAK2, ↑iNOS, ↑ Claudin 1 and 5, Occludin 1 and TLR4/MyD88/NF-κB p65 at 200 mg/kg only	↑ SCFAs ↑*Lachnospiraceae_NK4A136*, *Firmicutes*, *Clostridia*, *Lachnospiraceae*, *Lachnospirales*, *Lachnospiraceae_NK4A136* and *unclassified_f_Lachnospiraceae*	-
[69]	*Rhizopus nigricans* polysaccharide; 150 mg/kg	DSS (2%)	BALB/c mice, Sex n/i *n = 18*; 6/group	↑ Crypth depth ↑ Villus length ↑ Acid and mucus-secreting goblet cells	↑ *Lachnospiraceae_NK4A136_, Rikenella, Rosburia, Ruminococcus_1, Coprococcus_1, Prevotellaceae_UCG-001* and *Parabacteroides.* ↑ Total SCFA and lactate level	-
[70]	Polysaccharides; 300 mg/kg	TNBS (40 mg/kg)	Male Sprague-Dawley rats, *n = 18*, 6/group	↓ DAI ↓ Histopathological score	↑ Microbial diversity ↑ *Proteobacteria, Bacteroidetes* ↓ *Firmicutes*	↓ MPO
[122]	Alginate oligosaccharides; 200 mg/kg	–	Male & Female rats, strain n/i *n = 25*, 6/group	↑ Body weight ↑ Goblet cell per unit length	Male: ↑*Bifidobacterium, Bacteroides, Akkermansia, Anaerostipes, Anaeroplasma* and *Blautia* Female: ↑ *Bacteroides*	-

**APS3a**, non-honey-processed *Astragalus* polysaccharides; **COX-2**, cyclooxygenase 2; **Cy**, cyclophosphamide; **DAI**, disease activity index; **DSS**, dextran sodium sulfate; **F/B ratio**, Firmicute to Bacteroidete ratio; **GSH**, glutathione; **HAPS3a**, honey-processed *Astragalus* polysaccharides; **iNOS**, inducible Nitric Oxide Synthase; **IKK,** inhibitory kappa B kinase α; **n/i**, no-information provided; **MDA**, malondialdehyde; **MPO**, myeloperoxidase; **NO_3_/NO_2_**, nitrate/nitrite; **SOD2**, superoxide dismutase; **SCFA**, short-chain fatty acids; **TLR4**, toll-like receptors-4; **TNF-α**, tumor necrosis factor-alpha; **ZO-1**, zonula occludens 1.

#### Polysaccharides as modulators of the gut microbiota

Significant changes of polysaccharide therapy to gut microbiota composition and abundance can be seen in Table 3. Most polysaccharide therapy reported the restoration of *Firmicute* to *Bacteroidete* ratio (F/B ratio), whilst increased F/B ratio has been intrinsically related to colitis or dysbiosis, but F/B ratio should be approached with caution as both increase and decrease have been linked to dysbiosis for different causes [71–73]. Treatment with polysaccharide from apple was also able to restore the gut microbiota abundance similarly to the control group, showing profound treatment potential [67]. However, complete restoration of microbiota richness and diversity was not evident for every case, as polysaccharides from *Atractylodes macrocephala* herbs only partially restored microbiome abundance [65]. Notwithstanding this variable efficacy, where a positive outcome was demonstrated this invariably involved a reduction of inflammation-induced fecal and serum metabolites such as valine, propionic acid, tryptophan, 5-aminopentanamide, glycine, and histidine [65], suggesting a wider inhibition of pro-inflammatory mediators leading to a cumulative therapeutic effect.

Whether certain polysaccharide therapy can reverse colonic damage completely should be further studied, as the difference may lie in various factors such as the dose of polysaccharide, the origin of polysaccharide (taking into consideration natural variation), and finally the level of regulation of colon inflammation in the preclinical model being studied. Certain studies had opposite effect of *Firmicutes* abundance, with one increasing the abundance and the other decreasing. However, *Lactobacillus* and SCFA-producing microbiota, or SCFAs itself were generally increased throughout the studies [62,64,65,68]. Total SCFAs and lactate consistent with the changes in gut microbiota composition were also seen in animal model of colorectal cancer treated with *Rhizopus nigricans* polysaccharide, with increased abundance of *Lachnospiraceae_NK4A136*, *Rikenella*, Rosburia, *Ruminococcus_1*, *Coprococcus_1*, *Prevotellaceae_UCG-001*, and Parabacteroides bacteria [69]. SCFA-producing bacteria were also negatively associated with inflammatory cytokines, also alluding to its anti-inflammatory effect [64].

Interestingly, the number of side chains for a given polysaccharide seemed have an effect in regulating intestinal microbiota, with fewer side chains being more effective indicating a structure function relationship. Such effect was shown able to be attained through honey-processing polysaccharide. When processed with honey, polysaccharides reduced F/B ratio and significantly improved acetic acid and propionic acid concentration. Polysaccharides without being honey-processed also increased butyric acid concentration, which likely resulted from altered *Firmicutes* abundance [58].

### Anthraquinone (Emodin)

Emodin (1,3,8-trihidroxy-6-methyl-anthraquinone) is an anthraquinone compound found it in roots and plants such as *Aloe vera*, which has gained particular interest amongst researchers in relation to the impact of this class of natural products on inflammatory conditions [74]. Emodin is commonly used in the food industry as an additive that enhances product shelf-life. Recent studies have identified multiple biological actions for Emodin with beneficial activities including anti-inflammation, gut-immunity symbiosis, and enhancing anti-oxidation capacity, which are all relevant pathophysiological actions central for disease progression in IBD pathogenesis [31,32]. For instance, emodin prevents pseudorabies-virus-induced apoptosis in both *in vivo* and *in vitro* studies, implying its regulatory effect on the immune system, which can also be relevant to gut dysbiosis [32]. In support of this notion, an *in vivo* study showed a significant alleviation of inflammatory biomarkers such as cytokines IL-1β, IL-4, TNF-α, decreased colon mucosal permeability, all linked to improved gut microbiota, including enhanced *Verrucomicrobia* and *Firmicutes* in mice colons, while also exhibiting analgesic effects against neuropathic pain [31]. Other studies have also documented the toxicity effects of Emodin on kidney cells [75], identifying excessive endoplasmic reticulum (ER) stress coupled with mitochondrial dysfunction as a mechanism that leads to apoptosis in human T cells [76]. Due to these conflicting results, further study is required to determine whether emodin will be beneficial or damaging to the colonic tissues and ultimately its impact on IBD pathogenesis.

Moreover, it’s relevant to note that emodin consumed via the oral pathway opens the possibility of interaction with digestive factors, which may affect its conformation and thus its potential antioxidant/anti-inflammatory activity, leading to changes in its biological function and/or therapeutic actions [77]. In addition, conflicting information on edmodin metabolism is also reported; whether metabolites derived from emodin may also be biologically active is presently unclear. For example, trans-emodin dianthrones (TED) showed rapid absorption, wide distribution, and long half-life when administered by oral gavage [78]. However, the drug is rapidly metabolized further for excretion when administered through the intra-vascular (i.v) path. Evidence supporting this conclusion includes a change in pharmacokinetic criteria with the circulating half-life for oral gavage of emodin was determined to be 6.4 h, whereas this parameter fell to 1.8 h when administered via i.v. [78]. However, another study showed no significant difference between intraperitoneal injection and oral administration, but interestingly a significant difference in the rate of metabolism was noted between sexes, with female mice metabolizing emodin significantly faster than males [79]. Therefore, all the factors previously described including metabolism and potential interactions, dosage, route of administration, sex of the recipient, among other may yield variable biological activities and, further studies directly addressing this knowledge gap are warranted before emodin can be considered a useful adjunctive therapy for IBD patients.

#### Emodin as an anti-inflammatory agent

*In vivo* studies using animal models showed significant attenuation of pathological score defined by intestinal necrosis or hemorrhage histopathological in interventions with emodin despite the ongoing presence of significant inflammation, alluding to emodin’s protective mechanism against intestinal damage [80]. In an *E. coli* O_1_-induced diarrhea experimental model, mice treated with emodin for 7 days showed ameliorated colon shortening, reduced colon atrophy, increased mucosal goblet cell density, and improvement of tight junction barrier compared to the insult group in the absence of intervention. Additionally, emodin was shown to elicite significantly higher MUC-2 fluorescence intensity, indicating goblet cell preservation yielded continued mucin production, along with a reduced levels of IL-1β, IL-6, and TNF-α [80]. Another study reported that emodin prevented intestinal permeability by increasing zonula occludens-1 (ZO-1) and occluding expression – but not as effective as claudin-1 – in the intestine of septic mice [81]. Emodin (EMO)-loaded Poly (DL-lactide-co-glycolide)/Eudragit S100/montmorillonite nanoparticles (EMO/PSM NPs) showed comparable anti-inflammatory effect to 5-ASA, alluding its therapeutic potential [82]. Once again, further study is necessary to determine efficacy, toxicity, and side effects of nanoparticle delivery of emodin in human subjects.

#### Emodin as scavengers of reactive oxygen species (antioxidant capacity)

Emodin shows antioxidant activity as evidenced by significant reduction of MDA levels combined with enhancement of both low- (GSH) and high-molecular weight (SOD) antioxidants, signifying emodin’s potential to protect against oxidative stress through targeting different pathways linked to tissue redox modulation [83]. When delivered via nanovehicle, emodin also showed enhanced biological activities as judged by significant decreases in colonic MPO and nitric oxide (NO) [82]. This activity is likely explained by the chemical reduction of the quinone to a corresponding phenol by biological reductants. For example, *o*-quinones, a primary product, can be reduced by biological reductants to yield phenols and *o*-diphenols and then oxidized back to the corresponding *o*-quinones by tyrosinase [84]. Interestingly, the available evidence indicates that the *o*-diphenolic structure in the chemically reduced hydroxyanthraquinone molecules could markedly enhance the radical scavenging property of this natural product [85] with potentially the redox recycling of the active scavenger adding to the scavenger efficacy.

#### Emodin as modulators of the gut microbiota

Recently, emodin has been also documented to improve richness and diversity microbiota, significantly improvement richness and composition of *Bacteroidetes*, *Firmicutes*, *Verrucomicrobia*, and reduction of *Proteobacteria* in intestinal disorders [80]. Gao et al. [80] reported that emodin reestablished microbial content in the colon after being disrupted by pathogenic enterotoxigenic *Escherichia coli*, implying the capacity of emodin to suppress pathogenic microbiota and enhance growth of healthy composition and abundance. Beneficial effects of emodin on oxidative stress, inflammation and gut microbiota are summarized below (Table 4) and demonstrate that the bioactivity for this natural product involves the modulation of many disparate pathways.

**Table 4.** Mechanism of actions of emodins in alleviating IBD with reference sources cited in the far-left column.

Ref.	Active ingredient (where identified) and dose	Chemical stimulus	Animal	Bioactivity summary	Change in gut microbiota	Proposed antioxidant mechanism
[80]	Emodin; 8.75, 17.5 or 35 mg/kg	*E. coli* (2.5 × 10^11^ CFU/mL)	Male & Female Kunming mice; *n = 80*; 10/groups	↓ Pathological score ↓ IL-1β, IL-6, TNF-α and COX-2 ↑ sIgA and MUC-2	↑ Richness and diversity ↑*Bacteroidetes*, ↑ *Firmicutes* and *Verrucomicrobia*	↓ MPO
[81]	Emodin; 10, 20 or 40 mg/kg	CLP	Male BALB/c Mice, *n = 150*; 30/groups	↓ Intestinal injury score ↓ IL-1β, IL-4, IL-17, IL-2, IL-3, IL-10, TNF-α, IFN-*γ*, GM-CSF ↑ ZO-1 and occludin ↓ Caspase-3 and SIRT1	↓ *Proteobacteria* and *E.coli,* ↑ *Firmicutes* and *Bacteroidetes*	-
[82]	Roots and rhizomes of *R. hotaoense –* EMO/PSM NPs; 5 or 20 mg/kg	DSS (6 g/kg/d)	Male BALB/c mice, *n = 49*; 8/groups	↓ Clinical score ↓ Colon shortening and morphological damage		↑ GSH ↓ MPO
[83]	Emodin; 20, 40 or 80 mg/kg	CLP	Male BALB/c mice, n = 48; 8/group	↓ Sepsis score ↓ IL-6 and TNF-α		↑ GSH ↑ SOD ↓ MDA

**CLP**, cecal ligation and puncture; **COX-2**, cyclooxygenase-2; **DSS**, dextran sodium sulfate; ***E. coli***, *Escherichia coli*; EMO/PSM NPs, emodin-loaded poly (DL-lactide-co-glycolide)/Eudragit^Ⓡ^ S100/montmorillonite nanoparticles; **GM-CSF**, granulocyte macrophage colony-stimulating factor; **GSH**, glutathione; **IFN-*γ***, interferon gamma; **MPO**, myeloperoxidase; **sIgA**, secretory immunoglobulin A; **SIRT1**, Sirtuin 1; **TNF-α**, tumor necrosis factor-alpha; **ZO-1**, zonula occludens 1.

### Short-chain fatty acids (SCFA)

Short-chain fatty acids (SCFA) are byproducts produced by gut microbiota commonly categorized by valerate (number of carbons 5; C5) and the most abundant organic substrates: Butyrate (C4), Propionate (C3), and Acetate (C2) [33]. These SCFA are biologically active and have been widely recognized for their beneficial effects and their important role in gut health [8]. Dietary fiber supplements are often incorporated to increase SCFA production, but different fiber and intervention may lead to different effects on the microbial composition, diversity, and the corresponding individual immune response [34]. Interestingly, fiber supplementation with inulin (fructo-oligosaccharide) showed significant elevation of *Actinobacteria* abundance and suppression of *Bacteroidetes*, while supplementing isomalto-oligosaccharide fiber significantly increased *Bacteroidetes* and decreased *Firmicutes* in the microbiota population, without significant changes on total SCFA levels [34].

On the another hand, dietary choices play a critical role in modulating intestinal SCFA. Diets lacking in fiber exacerbate diseases in experimental models of intestinal injury and colorectal cancer [86]. Conversely, diets high in fiber are documented to protect against inflammatory diseases. For example, data produced by Laurence et al. underpin the protective effects of rich-fiber diet. Specifically, their study documented that fiber-derivated SCFA binds to GPR43 on colonic epithelial cells leading to the stimulation of a K(^+^)-ion efflux and cellular hyperpolarization, which promotes the activation of the NLRP3 inflammasome pathway, crucial for gut homeostasis [87]. Also, it has been reported that antenatal supplementation with a high-fiber diet leads to a reduction in intestinal inflammation amongst the corresponding adult offspring that are subsequently challenged in an experimental model of colitis [88].

Available evidence has shown that different SCFAs may exhibit varying biological effects depending on the specific change in level of the metabolite under different pathophysiological conditions. For instance, supplementing butyrate and propionate led to diminished proliferation and altered CD4^+^ and CD8^+^ T-cell populations; however, only butyrate reduced CD25^+^ T-cell expression in a dose-dependent manner. Under the same conditions, acetate showed no significant effect [89]. However, Vieira et al. [90] showed that acetate significantly suppressed inflammation by inhibiting pro-inflammatory pathways linked to the transcriptional activation of NF-κB and more broadly the degree of neutrophil activity, and instead promoted the production of anti-inflammatory TGF-β, annexin A1, and IL-10, thereby eliciting an anti-inflammatory activity. Furthermore, food-derived oryzanol (ORY) treatment, which was significantly linked to elevated fecal SCFA contents lowered *Firmicutes* abundance while improving *Bacteroidetes* abundance while not markedly altering butyric acid level [91]. Other authors have shown that butyrate synthesis and the bacteria responsible for producing this SCFA have been found to exert the highest anti-inflammatory effect on the gastrointestinal tract and most significant regulation of the immune response through inhibition of proinflammatory cytokines and promotion of anti-inflammatory cytokines such as IL-10 [92]. Thus metabolic synthesis of specific SCFA may need to be studied separately to accurately identify an individual biological effect.

Notably, SCFA have been shown to provide beneficial effects on gastrointestinal disorders through interaction with immune and epithelial cells in the colon tissue. G protein-coupled receptors (GPCRs) are not only highly expressed on enterocytes and enteroendocrine cells in the gastrointestinal tract but also in the macrophages, dendritic cells, lymphocytes, and neutrophils [93]. SCFAs bind to GPR41 and GPR43 (also known as free fatty acid receptors FFAR3 and FFAR2, respectively), triggering the differentiation and maturation of immune cells and thereby, acting as important mediators of the mature immune state. Both GPR41 and GPR43 receptors activation leads to the inhibition of adenylyl cyclase and, subsequently, decreases the levels of cyclic AMP (cAMP), extracellular signal-regulated kinase (ERK), and impacts transcriptional activation of the nuclear factor kappa-light-chain enhancer of activated B cells (NF-κB) cascade, which then collectively manifests as a decrease in colon inflammation [94].

Another possible mechanism by which SCFA could enhance intestinal barrier function and reduce inflammation is through histone deacetylase (HDAC) inhibition. Butyrate [86] and *Clostridium butyricum*-derived butyrate induce an increase of IL-10 expression and upregulate regulatory B cells *via* inhibiting HDAC1 activation. In addition, SCFA also impact macrophage polarization (that is the ratio M1 to M2 phenotype) through GPR43 activation, HDAC inhibition [95] and/or JAK/STAT3/forkhead box O3 (FOXO3) axis inactivation [96]. Specifically, butyrate induces the expression of synaptopodin, an actin-binding protein that enhances intestinal barrier function, via HDAC inhibition [97] and also to suppress the production of neutrophil-derived MPO and proinflammatory mediator production, perhaps through its HDAC inhibitory function [98]. Furthermore, butyrate may inhibit neutrophil migration and the consequent realizing of NETs in cells from CD and UC patients [98]. Thus this complicate of data reinforces the suppressive effect of SCFA on intestinal inflammation and oxidative stress leading to improved epithelial barrier function.

Numerous studies have shown that the pathogenesis of IBD and clinical evolution may be modulated by SCFA; however, the exact mechanism of action and linkage of all SCFA in IBD is still unclear [99]. For instance, the combination of *Veillonella ratti* (*V*. *ratti*) and *Lactobacillus acidophilus* (LA) leads to significantly elevated total SCFA levels and suppressed lactate, along with significantly ameliorated clinical and histological symptoms in experimental UC [100]. Overall, these outcomes imply a connection between high SCFA, microbiota, and IBD in humans. A link between polysaccharides and SCFA production in the gut has also been proposed. For example, α-galacto-polysaccharide derived from *Lupinus albus* is also a known prebiotic leading to increased SCFA through gut microbiota abundance [101]. Indeed, its treatment also significantly reduced DAI, improvement intestinal permeability, and goblet cell count but mild inflammation and mucosal damage were still seen [90]. In addition, *Lactobacillus plantarum* ZJ316 therapy, which showed significantly higher level of SCFA compared to mesalazine, both treatments successfully restored colon weight, clinical markers and IL-1β, IL-8, and TNF-α inflammatory cytokines compared to the DSS (insult) group, however, ZJ316 therapy was more effectively in suppressing IL-6 expression in rodents [102].

Along with butyrate, both propionate and acetate are SCFA metabolites derived from gut microbiota. Butyrate, as shown in the section above, is a more well-known SCFA and has shown evidence as a therapeutic agent for IBD. However, studies have also alluded to possible therapeutic effects of other SCFAs including the protective action of propionate and acetate on colon pathophysiology. After all, SCFAs seem to be tightly related to the colon, as seen in the distribution of olfactory receptor 78 (Olfr78), an SCFA receptor expressed by the enteroendocrine cell, commonly expressed in cecum, distal large intestine, and rectum [103]. The specific affinity of both propionate and acetate to Olfr78 receptor may promote the secretion of the anorexigenic gut peptide YY (PYY) [104], a hormone involved in the maintenance of gastrointestinal function. Indeed, PYY has been shown to be significantly lowered in the colon of patients with inflammatory bowel syndrome, implying its contribution to clinical symptoms [105]. Thus propionate and acetate may have a major role in inhibiting inflammation and oxidative stress as well. Furthermore, *Moringa oleifera* leaves which resulted in elevated acetic, propionic, and butyric acid, in descending order, led to significantly decreased total polyp burden and reduced proinflammatory chemockine monocyte chemoattractant protein 1 (MCP-1), while increased more than two times IL-10 [106]. However, further identification of SCFA and their corresponding bioactivity is necessary, before concluding that widespread SCFA supplementation is suitable as a therapy for the maintenance of remission in patients diagnosed with IBD.

**Figure 2.**
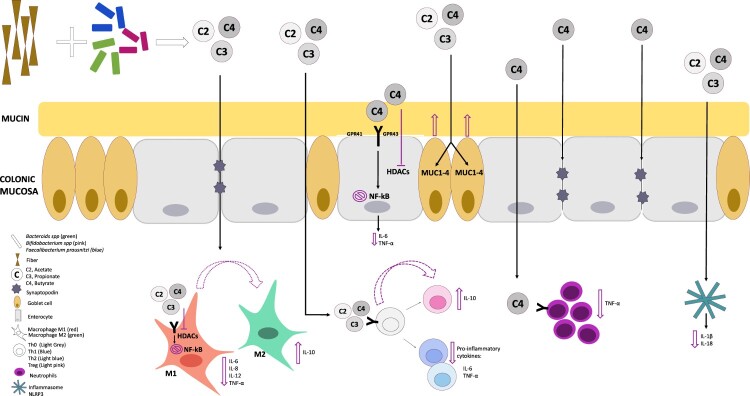
Summary SCFA bioactivity on various intestinal and immune cell types. Dietary fiber is fermented by gut microbiota, leading to the production of SCFAs. SCFA are ligands for receptors on colon epithelial cells of the colonic mucosa and immune cells such as macrophages, lymphocytes, and neutrophils. Specifically, butyrate interacts with intestinal synaptopodin, to aid wound healing and intestinal permeability through the suppression of histone deacetylase (HDAC) and NF-kB. SCFAs are also associated with macrophage polarization towards anti-inflammatory M2 macrophages, which increases the secretion of anti-inflammatory IL-10 cytokine. SCFA interacts with T-cell proliferation and activation, enhancing T regulatory cells and suppressing pro-inflammatory T cells, along with modulate inflammasome.

Given the proximity of the gut epithelium to the gut microbiota, multiple pathways downstream of SCFA sensing have evolved to promote gut barrier function; these multiple activities are shown in Figure 2.

### SCFA – butyrate (C4)

#### Butyrate as anti-inflammatory agents

In mice, butyrate oral administration led to significant reduction in disease severity and histopathological signs in IBD such as immune cell infiltration in colon, and promoted autophagy [12,98,107,108]. Moreover, other studies have documented that butyrate partially reduced inflammation in mice through significant inhibition of CitH3 and thus, potentially acting via the inhibition of NET formation [98]. On the other hand, it has been observed that butyrate reversed the depletion of F4/80+ macrophages induced by clodronate liposomes – a common inducer of macrophage apoptosis – in mice insulted with DSS, leading to a significant improvements in goblet cell viability and mucus secretion from the colon mucosa [12].

Furthermore M2 (resolving) macrophage-associated genes and levels of p-STAT6 phosphorylation in F4/80+ macrophages were significantly promoted after supplementing B-hydroxybutyrate (BHB) and this activity was markedly suppressed by the pharmacological agent AS1517499, which inhibits STAT6 phosphorylation [12,108], implicating the STAT6 pathway in the biological action of butyrate. In summary, mice studies showed significant effect of butyrate in lowering DAI and histopathological markers of colon damage with significant promotion of the macrophage M2 phenotype that drives repair and resolution of the inflamed colon.

Butyrate administration in human studies showed significant reduction in release of neutrophil-mediated proinflammatory cytokines and suppression of IBD biomarkers such as calprotectin (CP) and LCN2 (lipocalin 2) [98]. Supplemented patients identified with a remissive UC condition also had significantly lower macrophage number than active UC patients, along with a markedly M2-like immunophenotype within the colon lamina propria [12]. The improvement of butyrate’s treatment in mucus barrier damage was also noted due to the increasing expression of *MUC2* and *SPDEF* (SAM pointed domain containing ETS transcription factor) [12], which are linked to improved mucosal function.

Overall, butyrate therapy leads to significantly lower disease severity and inflammatory cytokine level with macrophages having a role in its mechanism of action in studies using animal models and in human trials. Following BHB therapy, both humans and mice, displayed significant reduction in colonic tissue protein expression of *ACAT1*, *HMGCS2*, *BDH1* which are genes commonly expressed in active IBD [109]. However, there was no significant difference in alleviating intestinal permeability (ZO-1, occluding) for BHB treated group compared to control group irrespective of whether studies were conducted with humans or a using mouse model. Interestingly, BHB significantly alleviated DSS-induced inflammatory signs but had no effect without DSS induction, implying its therapeutic effect on only the injured tissues [109]. This conclusion may lead to the deduction that butyrate therapy is only effective in damaged tissues. However, another study showed that butyric acid only showed significant elevation in a healthy group, while significant enhancement of the butyryl-CoA:acetate CoA-transferase pathway, which modulates endogenous butyrate-production in the colon, was noted in this experimental disease model group [101]. This leads to the question of whether modulation of the butyryl-CoA:acetate CoA-transferase pathway and utilization of increased butyric acid depend on inflammatory process and mucosal damage to drive the enhanced production of this SCFA. However, in direct contrast with this notion, supplementing with sodium butyrate showed no significant difference in disease-induced weight loss in a mouse model of IBD [107]. Overall, a pure anti-inflammatory role for butyrate may not be the sole mechanism of action, but rather the extent of bioactivity may show some dependence on healthy or disease state and whether interaction with colonic macrophages occurs at a suitable timeframe during the pathogenesis of the disease stage.

#### Butyrate as scavengers of reactive oxygen species (antioxidant capacity)

Several studies have identified that butyrate also displays antioxidant activity. For example, butyrate administration led to decreased ROS production, accumulated lipid oxidation (MDA), and neutrophil-MPO levels while increasing tissue SOD and GSH levels in mice [98]. An increase of L-012 intensity, a luminol-based chemiluminescent probe used to detect ROS and reactive nitrogen species (RNS), was also observed in an experimental model, while a less intense abdominal signal observed in mice treated with sodium butyrate which was consistent with decreased amounts of MPO, an enzyme which produces ROS [107]. In addition, sodium butyrate along with 5-ASA treatment significantly reduced lipid peroxidation and restored GSH, and induced Nrf-2 activation leading to enhanced HO-1 expression [107]. In conclusion, butyrate plays a significant role in attenuating oxidative stress in IBD.

#### Butyrate as modulators of the gut microbiota

As have been mentioned, SCFA are metabolites derived from microbiota and can alter microbial composition [110]. Most importantly, a human study showed that IBD in remission displayed a significant increase in fecal butyrate and microbiome known to produce butyrate, showing positive association between the alleviation of IBD and butyrate production [12].

In mouse models of IBD, treatment with butyrate showed no significant effect in altering microbial α-diversity. However, supplemented butyrate led to increased abundance in strains such as *Firmicutes* and *Lachnospiraceae* and changes in their relative composition [102,109]. Indeed, butyrate-producing bacteria successfully elevated Dact3 expression, which interacts with c-Jun N-terminal kinase (JNK) pathway and was associated with the alleviation of disease severity inflammatory biomarkers, result that non-butyrate producing microbiota was unable to replicate [108]. Taken together these data show that endogenous butyrate levels strongly depend on the microbiome phenotype and its capacity to sustain butyrate production.

Another study discussing the mixture between *V. ratti* and L. acidophilus showed that this dietary mixture significantly elevated SCFA levels and lowered the *Firmicutes/Bacteroidetes* ratio [100]. Treatment with a mixture of *Lupinus albus* α-galacto-oligosaccharides (LA-GOS) also elevated *Bacteroidetes* and *Firmicutes* abundance and suppressed the relative proportion of *Proteobacteria* abundance [101]. In the DSS colitis model, intervention with *Lactobacillus plantarum* ZJ316, which utilizes butyric acid, showed a direct relationship with *Faecalibacterium*, *Agathobacter*, and *Firmicutes* and negative association with *Bacteroidetes* and *Actinobacteria* [102]. Interestingly, *Faecalibacterium*, *Agathobacter*, unidentified_*Lachnospiraceae*, and *Firmicutes* which were associated with butyric acid, showed inverse relationships with inflammatory cytokines, further reinforcing butyric acid’s capacity to alleviate inflammation [102]. Under the same experimental conditions, Bacteroidetes showed a negative association with butyric acid, and was linked to increased levels of inflammatory cytokines [102], suggesting that the relationship between butyrate and microbiota, is critical to ameliorating inflammatory process in the gut. However, a different study showed that butyrate therapy post antibiotic treatment alleviated disease severity, implying butyrate’s independent effect from gut microbiota while butyrate’s dependence on macrophages was also evident [109]. Thus there seem to be conflicting results in the relationship between butyrate and gut microbiota while more consistent evidence exists for butyrate bioactivity in promoting phenotypic change in colonic macrophages. Overall, the role of SCFA butyrate in promoting intestinal epithelial barrier function and regulating the host mucosal immune system in intestinal injury through the regulation of oxidant and inflammatory pathway and microbiota modulation are outlined in Table 5.

**Table 5.** Mechanism of actions of butyrate in alleviating IBD with reference sources cited in the far-left column (to be continued)

Ref.	Active Ingredient (where identified) and dose	Chemical stimulus	Animal	Bioactivity Summary	Change in gut microbiota	Proposed antioxidant mechanism
[89]	Butyrate; 0–1.6 mM	**-**	UC patients *n = 28*	**T cell treated with Butyrate:** ↓ CD4+, CD8+ T cell proliferation ↓ T cell activation, ↓ CD25+ cells ↓ HDAC I/II	-	-
[98]	Butyrate; 200 mM	DSS (2.0%) mice only	IBD patients *n = 187*; *n = 112* active CD, *n = 43* active UC, *n = 32* HC Male C57BL/6 mice, *n = 48;* 12/group	**CD and UC patients**: ↓ IL-6, IFN-γ, TNF-α, IL-17A, IL-22, IL-8, S100A8/9 and LCN2 ↓ Neutrophil migration **Mice:** ↓ Body weight loss, ↓ Colon shortening, ↓ Pathological disease score, ↓ IL-6, TNF-α, INF-γ, CXCL1, S100A8/9, LCN2 and CitH3.	-	**CD and UC patients**: ↓MPO **Mice**: ↓ROS
[12]	Butyrate; 20 mg/kg	DSS (3.0%) mice only	UC patients *n = 28;* active and inactive UC Male BALB/c mice, *n = 24*; 6/group	**Inactive UC patients:** ↑ Butyrate level ↓ Macrophage expression ↑ Arg1+ (M2-like immunophenotype) **Mice:** ↓ Body weight loss ↓ DAI ↓ Epithelial damage ↓ Goblet cell loss ↓ Leukocyte infiltration and F4/80+ macrophages. ↑ MUC2 and SPDEF expression.	**Inactive UC patients:** ↑*Lachnospirceae* ↑*Ruminococcaceae*	**Inactive UC patients:** ↓ iNOS ↑CD206/iNOS
[109]	BHB ketone; 15 mg/25 g	DSS (2.5%) mice only	IBD patients (active and remission CD and UC);*n = 112* Male C57BL/6J mice, *n = 20–40*; 7–10/group	**Active IBD patients:** ↓ ACAT1, HMGCS2, BDH1. **Mice**: ↓ Body weight loss ↓ DAI and colon shortening ↓ Histology score ↑ IL-4Ra, IL-10, Arg-1, Chil3 ↑ F4/80+CD206+ M2 macrophages mRNA expression ↑ *p*-STAT6 in F4/80+ macrophages.	**Mice**: ↑Firmicutes ↑Lachnospiraceae ↓Lactobcillaceae	-
[107]	Sodium butyrate (NaB); 0.1 M or 500 g/kg/day	DSS (2.5%)	Male C57BL/6 mice, *n = 44*; 8-12/group	↓ Mortality rate ↓ DAI\ and colon shortening ↓ Histopathology score ↓ Inflammatory cell infiltration ↓ Tissue damage, ↓ NF- κB/NLRP3 ↑ Pink1/ Parkin axis ↑ LC3 II ↓ p62*, ↑ COX-2	-	↓ L-012 luminescence intensity ↓ Lipid peroxidation products ↑ GSH level ↑Nrf-2/ HO-1

**ACAT1**, acetyl-CoA acetyltransferase 1; **Arg-1**, arginase 1; **BHB**, ketone body B-hydroxybutyrate; **BDH1**, 3-hydroxybutyrate dehydrogenase I; **Chil3**, chiinase-like protein; **CD:** Crohn’s disease; **CXCL-1**, Chemokine ligand 1; **CitH3**, Citrullinated H3; **COX-2**, Cyclooxygenase-2; **DSS**, Dextran sodium sulfate; **DAI**, Disease Activity Index; **HC**, healthy volunteers; **HDAC**, histone deacetylases; **HMGCS2**, 3-hydroxymethylglutaryl-CoA synthase 2; **IFN-*γ***, Interferon gamma; **MPO**, Myeloperoxidase; **NF**-**κB**, Nuclear factor kappa B; **NLRP3**, NOD-like receptor protein 3; **LCN2**, lipocalin-2; **LC3**, Microtubule-associated protein 1A/1B-light chain 3; **TNF-α**, Tumor Necrosis Factor-alpha; **UC**, Ulcerative colitis.

### SCFA – propionate (C3)

#### Propionate as anti-inflammatory agents

Anti-inflammatory effect of propionate has been documented in studies focused on different types of gut microbiota, diet, and in direct supplementation of isolated cells. Propionate may be indicative of inflammation, or propionate-producing bacterium are strongly linked with the elevation of colon inflammation and intestinal damage [111]. For example, *P. freudenreichii*, which has been shown to enhance the production of mucin, is known to produce propionate as its most prominent metabolite. The administration of both live and supernatant derived from cultured *P. freudenreichii* displays a similar capacity to stabilize goblet cells exposed to inflammatory challenge, indicative of the anti-inflammatory effect of propionate [111]. Consistent with this result mentioned above, MUC2 protein secretion, which is a major component of intestinal mucin, was significantly elevated by both supernatant and live *P. freudenreichii* in DSS-treated mice compared to DSS-insult alone. This mucin phenotype was linked with decreasing levels of TNF-α, IL-6, IL-1β, and tendency of increase IL-10 production [111]. Moreover, natural compounds have shown a significant increase of butyrate and propionate levels in cecal and fecal content from mice insult with DSS, which are correlated with ameliorate colon inflammation [106].

However, not all available data is supportive for a colon protective role for propionate. Thus propionate’s biological effect may be questioned when delivered by supplementing in the diet. For instance, rats fed on cured meat, which had 18% higher proportion of propionate than comparable amounts of fresh meat, did not lead to a significant difference in CRP levels, inflammatory cytokines such as IL-6 and pro-inflammatory TNF-α, which did not increase above the limit of detection in the absence or presence of propionate [112]. A simple explanation for this lack of bioactivity may be that an 18% higher load of dietary propionate was not sufficient to yield a biological difference, albeit that normal dietary propionate intake in humans is commonly less than the level tested in this study [112].

#### Propionate as scavengers of reactive oxygen species (antioxidant capacity)

In colon treated with *Moringa oleifera* which showed a significant increase in both acetic and propionic acids, a parallel and significant increase in NADH-quinone reductase (NQO1) activity (2.5-fold) and glutathione-S-transferase (GST) (1.5-fold) activity was determined in the colon. Parallel decrease of colonic neutrophil recruitment by 2.5-fold was also detected, with a concomitant 20% decrease in MPO concentration leading to 2.3-fold lower detection of the lipid oxidation biomarker MDA in colon tissues, which is consistent with a decrease in oxidative damage in these tissues. These results may suggest a close relation between propionic acid production and alleviate of oxidative stress [106].

#### Propionate as modulators of the gut microbiota

Although reduction in inflammation was observed, propionic acid may encourage pathogenic microbiota growth. For instance, Ormsby et al. [113] have showed that propionic acid encouraged anaerobic biofilm formation, which are considered pathological in the sense that these structures are highly resistant to therapies to modulate bacterial growth and can lead to skewing microbiota populations [114]. Interestingly, it was seen that propionic acid was unable to alter LF82 (a strain of adherent-invasive *E. coli* (AIEC) infection) in ileum and large colon, however, pre-exposure of AIEC to propionic acid combined with exogenous propionic acid supplementation promoted colonization and long-term persistence [113]. This calls into question whether propionic acid therapy will contribute to aiding survival of pathogenic microbiota due to a nutritional effect, which may lead to an exacerbation of colon inflammation. In fact, LF82 which infected PA-fed mice was significantly more competitive compared to water-fed mice [113]. Although propionic acid may have antimicrobial properties, adaptability of microbial strains should be considered.

As has been mentioned previously, cured beef compared to fresh beef displayed 18% higher propionate level and 25% lower butyrate level. Rats treated with cured beef exhibited significantly higher operational taxonomic unit richness and significant difference in microbial composition compared to rats fed on fresh beef. Additionally, rats fed beef had significantly higher cecal levels of valerate (+10.3%), total branched chain fatty acids (BCFA, +16.3%), *iso*-butyrate (+14.8%) and trend of higher *iso*-valerate levels (+17.8%) compared to rats on the chicken diets [112]. However, total SCFA (including acetate) did not show a significant difference based on the group comparisons, which may imply propionate as the prominent bioactive metabolite that is involved in colon protective actions [112]. Interestingly, even though the alteration of propionate level was observed, there was no significant change in abundance for *Lactobacillus* [112], strains that have shown to significantly produce SCFA [115]. Other nutritional factors have to be investigated to detect microbiota alteration. For example, in this study the salt content in the cured meat may explain why *Lactobacillus* was not significantly affected.

Most relevant changes of propionate and its impact on intestinal inflammation are shown in Table 6.

**Table 6.** Mechanism of actions of propionate in alleviating IBD with reference sources cited in the far-left column (To be continued).

Ref.	Active Ingredient (where identified) and dose	Chemical stimulus	Animal	Bioactivity Summary	Change in gut microbiota	Proposed antioxidant mechanism
[113]	Butyrate; 200 mM	*E. coli* strains (1 × 10^9^ CFU)	Male C57BL/6 mice, *n = 24;* 4/group	–	↑ Adhesion and invasion, ↑ Biofilm, ↑ Acid tolerance, ↑Persistence in ileum and colon	–
[112]	Cured chicken or beef; containing 20 g/kg nitrite salt and 0.5 g/kg sodium ascorbate.	–	Male Sprague-Dawley rats, *n = 40;* 8/group	**Cured meat:** ↑ Urine production, ↓ Mesenteric fat, ↓ Retroperitoneal fat, ↑ PCC in rats.	**Cured meat (mainly Beef):** ↑ BCFA, ↑Valerate, total iso-butyrate, carbon disulfide, indole and cresol ↑OTU, ↑18% propionate, ↓25% butyrate.	**Cured meat (mainly Beef):** ↓Oxidative stress ↑stomach 4-HNE ↑GSH-Px.

BCFA, branched-chain fatty acids; CFU, Colony-forming unit; GSH-Px, Glutathione peroxidase; 4- HNE, 4-hydroxynonenal; OTUs, Operational taxonomic units; PCC, Protein carbonyl compounds.

### SCFA – acetate (C2)

#### Acetate as an anti-inflammatory agent

Supplementing acetate or acetate-producing microbiota is documented to ameliorate inflammatory response, leading to preserved body weight, lower mortality, lower disease severity, less shortening of colon, amelioration of intestinal permeability or barrier, lower histopathological score, lowered inflammatory cell infiltration, anticancer activity, and inhibited cell damage and death in experimental animal models [116–119]. Furthermore, IL-1β, IL-13, and TNF-α were also significantly inhibited by added acetate [116].

The increase of acetate production and its potential beneficial effect on inflammation can be mediated by fiber intake. For instance, when acetate was administrated in mice with a deficiency fiber diet, the suppression of disease severity, neutrophil level, and body weight loss but not colon shortening was observed [120]. Significant suppression of IL-1β release was also observed in mice that received oral *Christensenella minuta* DSM 22607, which produce 5:1 ratio of acetate and butyrate, but no significant effect on weight gain [121]. There may be factors influencing these results. For instance, alginate oligosaccharide administration – a source of dietary fiber – led to significantly increased weight in male rats compared to female rats, which may allude to sex difference in AOS effectiveness [122]. An elevated total SCFA content and an increased microbiome abundance/diversity in the presence of butyrate and acetate, but not propionate, were seen in males compared to females [122]. Pre-existing colonic damage may be a significant factor as well, as dichloroacetate showed no significant changes in healthy colon, indicating its therapeutic action may be contained only to damaged colon [116]. Besides, dichloroacetate did not lead to attenuation of nuclear factor of activated T cells 1 (NFATC1), NLRP3 inflammasome, NF-κB nuclear fraction, cleaved caspase-1 expression in normal healthy mice. However, in experimental colitis in mice significant attenuation of proteins mentioned above was seen [116]. Furthermore, more researchers have mentioned different treatments such as Zearalenone – an estrogenic mycotoxin – has a significant impact in increasing the abundance of SCFAs producing bacteria as well as the fecal acetate content, along with suppressing Ras/Raf/ERK/cyclin D1 pathway [118].

#### Acetate as scavengers of reactive oxygen species (antioxidant capacity)

As acetate displays anti-inflammatory action, anti-ROS action was expected as well. Indeed, biomarkers of inflammation and oxidative stress were significantly attenuated by dichloroacetate, including NO and MPO [116]. MPO was also significantly lowered in mice treated with *L. acidophilus* and *C. minuta*, likely due to suppression of LCN-2 and cytokines such as IL-1β in the colon [121,123]. There are limited data to demonstrate antioxidant effect of acetate; however, it is possible to suspect that the reduction in inflammatory cytokines level may also contribute to reducing oxidative stress in the same local tissue environment.

#### Acetate as modulators of the gut microbiota

Besides alteration on inflammatory pathways and oxidative stress, significant changes to gut microbial composition were seen in therapies that significantly affect acetate levels, which can be found in Table 7. Amino acids such as tryptophan and phenylalanine showed association with gut microbiota, which call into question whether the effects are due to acetate itself or an accumulation of multiple factors [124]. In support of this point*, L. bacillus* shows direct relationships with the elevation of endogenous acetate, glycine, and aspartic acid, and *Prevotella* is linked with the elevation glycine and aspartic acid [123], hence any biological action cannot be ascribed to acetate alone.

**Table 7.** Mechanism of actions of acetate in alleviating IBD with reference sources cited in the far-left column (To be continued).

Ref.	Active ingredient (where identified) and dose	Chemical stimulus	Animal	Bioactivity summary	Change in gut microbiota	Proposed antioxidant mechanism
[116]	Dichloroacetate; 100 mg/kg	Topical Oxazolone (3%) → Enema oxazolone (1%)	Male BALB/c mice; *n = 20;* 3-5/group	↓ Histopathological score and disease activity index ↑ Colon length ↓NLRP3, NFATC, NF-κB and caspase-1 ↓ IL-1β and IL-13	-	↓MPO ↓NO
[117]	Acetate enema; 10 mM	DSS (2.5%)	Male C57BL/6 mice; *n = 10;* 5/group	↓ DAI ↓ Mucosal break ratio	-	-
[120]	Sodium acetate; 200 mM	DSS (0.5–2.5%) No Fiber	Male C57BL/6J mice; *n = 20;* 5/group	↓ DAI ↑ Body weight ↓ Colon length ↓ Blood neutrophils ↓ CXCR2	-	-

**CXCR2**, Interleukin 8 receptor; **DSS**, Dextran sodium sulfate; **DAI**, Disease Activity Index; **MPO**, Myeloperoxidase; **NFATC**, Nuclear factor of activated T cells; **NF**-**κB**, Nuclear factor kappa B; **NLRP3**, NOD-like receptor protein 3; **NO**, Nitric oxidase; **NLRP3**, NOD-like receptor protein 3.

Zearalenone, which showed a significant anti-inflammatory response, also led to a significant increase in unidentified *Ruminococcaceae*, *Parabacteroides*, *Blaustia*, which are known as SCFA producing bacteria. Consequently, significantly elevated fecal acetate was detected, and fecal acetate showed inverse relationship with smaller tumors (<2 mm), implying anticancer quality of acetate [118].

However, the therapeutic response may be associated with the capacity to respond to altered gut microbiota or endogenous SCFA production. In support of this idea, an increase of goblet mucus-secreting cells occurs in parallel with increased abundance of *Verrucomicrobiota*, *Bifidobacterium*, *Anaerostipes*, *Anaeroplasma*, *Blautia*, and *Akkermania* in male rats compared to female rats after alginate oligosaccharide therapy, along with a significant parallel increase in acetate and butyrate compared to female rats [122]. This may imply that the extent of therapeutic response may depend on an individual’s respond to SCFA producing therapy, as male rats showed significantly enhanced therapeutic response when acetate and butyrate production increased in parallel.

## Probiotics

Probiotics may be an essential therapeutic agent that can modulate the host–microbe interaction leading to potentially beneficial outcomes for IBD patients, which can be used both as a single agent and an adjunctive supplement conventional therapeutics [125]. Probiotics, along with prebiotics and symbiotic, are dietary supplements which can produce a synergistic effect when taken together. Prebiotics, which usually consists of non-digestible fiber, is a nutritional source for probiotics through active fermentation in the GI tract that yields several benefits for the host including promotion of selective bacterial growth, which may confer a health benefit [126]. Flavonoids and fiber can be considered prebiotics, as described above. At the same time, synbiotics are a combination of probiotics and prebiotics. The notion to induce synergistic effects by combining pro and prebiotics is commonly formulated with a combination of *lactobacillus GG* or *Bifidobacteria*, and inulin or oligosaccharides in a range of proportions and these formulations in IBD [127].

A meta-analysis of randomized controlled trials revealed both fecal microbiota transplant (FMT) and VSL#3 (a mixture of probiotics, including strains of *lactobacilli*, *bifidobacteria*, AND *Streptococcus salivarius*) showed beneficial therapeutic response in UC with significantly more patients achieving clinical remission compared to placebo controls. Outcomes from this review also highlighted fewer serious side effects from VSL#3′s compared to FMT treatment [128]. In terms of mechanism of action, the available data shows significant reduction of TNF-α, IL-1β, IFN-γ, and significant elevation of IL-10 in pouchitis of the ileal reservoir after VSL#3 therapy implying a specific probiotic effect in the ileal pouch [129]. Similarly, VSL#3 showed marked efficacy for post ileo-anal pouch surgery or antibiotic-induced remission of pouchitis [130]. Indeed, when VSL#3 adjuvant therapy was added to balsalazide (as a conventional anti-inflammatory IBD drug), significantly more patients (*p* < 0.02) were able to achieve remission and improve their endoscopic, clinical, and histology scores compared to treatment with balsalazide alone, including attaining remission significantly faster compared to the conventional treatment [131]. More recent evidence has shown the clinical benefit of combining VSL#3 (prescribed a dose of 3.6 × 10^12^ CFU [132,133]) with primary yields improved rates of remission and clinical score (UC Disease Activity Index; UCDAI) for UC patients compared to conventional therapy alone. However, despite these documented positive outcomes, the effectiveness of probiotics as a potential treatment for UC remains uncertain. For instance, another review of randomized controlled trials showed a very low certainty that probiotics administered either alone or combined with 5-ASA, help to prevent clinical relapse and maintenance of remission [134]. Similarly, the current guidelines for IBD management in children do not recommend probiotic adjuvant therapy for CD and furthermore, is unclear on whether VSL#3 or *E. coli* Nissle 1917 is beneficial for the treatment in children with UC [135]. Alternatively, VSL#3 together with antibiotic therapy has shown therapeutic benefits in the prevention of post-operative recurrence of CD [136]. On balance it is reasonable to conclude that the current body of available evidence is conflicting and does not provide a clear pathway to develop guidelines on the use of probiotic therapy for IBD.

Contemporary approaches that may improve the use of probiotics for clinical application include probiotic delivery with nanoenzyme coating therapy. Testing of this novel probiotic form led to significant improvement in weight loss, epithelial cell apoptosis, which subsequently enhanced MUC2 expression levels, tight junction protein interactions, and decreased DAI in an animal model of IBD [35]. In addition, *in vivo* studies using mesalamine loaded with probiotics showed significant restoration of body weight, fecal consistency, and decreased fecal bleeding [36]. Below, we briefly discuss the anti-inflammatory, gut microbiota restorative, and anti-oxidative effects of probiotics; more details are shown in Table 8.

**Table 8.** Mechanism of actions of probiotics in alleviating IBD with reference sources cited in the far-left column (to be continued)

Ref.	Active ingredient (where identified) and dose	Chemical stimulus	Animal	Bioactivity Summary	Change in gut microbiota	Proposed antioxidant mechanism
[35]	Pt-Lipid@EcN; dose n/i	DSS (3%)	Sex unspecified, C57BL/6 Mice; *n = 15;* 4 group	↑ Body weight ↓ DAI ↑ Colon length ↓ Histopathology score ↓ IL-6, TNF-α and IL-1β ↑ Occludin-1 and ZO-1	-	↓ MPO activity
[36]	Mesalamine and *Lactobacillus acidophilus* microparticles (F12);23 mg/kg/day	DNBS (15 mg/kg)	Female and Male Wistar rats: *n = 30;* 6/group	↓ Body weight loss, ↑ Stool consistency, ↓ Lesion score ↓ Macroscopy score	-	↑ GSH ↓ MPO ↓ LPO
[96]	7-mix strains (*E. hirae, L. casei, S. salivarius, F. prausnitzii, A. muciniphila, C. butyricum, L. salivarius*), mix-sup or hu-FMT; 1×10^8^ CFU per strain and 0.1 mL/10 g of body weight for hu-FMT.	DSS (3%)	Male BALB/c Mice; *n=30*; 6/group	↑ Body weight ↓ Colon shortening ↓ Histology score ↑ Occludin, ZO-1 and Muc2 ↓ IL-6, IL-1β, IL-12 and TNF-α ↑ IL-5 ↓ M1 (CD86+) for mix-sup only ↑ M2(CD206+) macrophages for mix-sup and 7 mix only. ↓ JAK/STAT3/FOXO3 for mix-sup and 7 mix only.	**Mix-sup and 7-mix:** ↑ α-diversity ↑ A. muciniphila ↑ L. salivarius ↑ F. prausnitzii ↑ Acetic acid ↑ Propionic acid ↑ Butyric acid ↑ Valeric acid ↑ *Lactobacillaceae, Lactobacillus, Lachnospiraceae, Lactobacillus murinus* in mix-sup ↑ Bacteroidales in the 7-mix group	↓ MPO ↓ iNOS for 7-mix and mix-sup only.
[100]	Butyrate-producing *Veillonella* and *lactobacillus;* 1 × 10^9^ CFU mL^−1^	DSS (2.5%)	Male C57BL/6 mice; *n = 60*; 12/group	↓ Body weight loss ↓ DAI score ↓ Fecal occult blood ↓ Colon shortening ↓ Histological scores ↑ Occludin	↑ Acetic acid ↑ Isobutyric, ↑ Total SCFA ↓ Lactate ↑ *Ligilactobacillus* ↑ *total Lactic acid bacteria* ↑ *Verrucomicrobiota* ↑ *Akkermansia* ↑ *bacteroides*	↑ SOD ↑ GSH ↓ MDA ↓ MPO
[102]	*Lactobacillus plantarum* ZJ31; 2.5 × 10^9^ CFU mL^−1^	DSS (2.5%)	Male BALB/c mice; *n = 25*; 5/group	↑ Colon weight ↑ Colon length ↓ Histological score ↓ IL-6, IL-8, IL-1β and TNF-α	↑ *Faecalibacterium* ↑ *Agathobacter*, ↑ *Roseburia.* ↑ *Firmicutes* ↓ *Bacteroidetes* ↓ *Actinobacteria* ↑ *Intestinimonas* ↑ *Butyricoccus* ↓ *Paracoccus* ↓ *Erysipelatoclostridium* ↓ *Acinetobacter* ↓ *Luteimonas* ↑ Acetic acid ↑ Propionic acid ↑ Isobutyric and Butyric acid ↑ Valeric acid	-
[108]	Butyrate-producing *Faecalibacterium prausnitzii* A2–165; 1 × 10^9^ CFU	DNBS (200 mg/kg)	Male C57BL/6 mice; *n = 24;* 8/group	↑ Dact3 ↓ Body weight loss ↓ IFN-γ, IL-6, IL-17A, MCP-1	*-*	↓ MPO activity
[111]	*Propionibacterium freudenreichii;* ‘LPF’; 1 × 10^8^ CFU and ‘SPFC’; 1 mL	DSS (5%)	Male Sprague-Dawley rats; *n = 30;* 6/group	**SPFC:** ↓ Histopathology score ↑ Goblet cell and Mucin **LPF:** ↑ Body weight change ↓ DAI ↓ Histopathology score ↑ Goblet cells and MUC2 level ↓ TNF-α, IL-6, IL-1β	**SPFC:** ↑ Acetate ↑ Propionate ↑ Butyrate **LPF:** ↑ Acetate ↑ Propionate ↑ Butyrate	-
[121]	Acetate-producing bacteria *Christensenella minuta*; 1 × 10^9^ CFU/mL	DNBS (175 mg/kg)	Male C57BL/6J mice; *n = 40;* 10/group	↓ Macroscopic and microscopic score ↓ Colon weight ↓ IL-1β	**-**	↓ MPO
[123]	*Lactobacillus acidophilus* KBL402 and KBL409; 1 × 10^9^ CFU	DSS (2%)	Female C57BL/6J mice; *n = 32;* 8/group	↑ Body weight change % ↓ DAI ↑ Colon length ↓ Histological score ↓ FN-γ, IL-1β, IL-4, IL-6, IL-17A, TNF-α	↑ *Akkermansia* ↓ *Bacteroidetes* ↓ *Mucispirillum* ↑ *Prevotella*	↓ CCL2 ↓ CXCL-1 ↓ MPO
[139]	*Lactobacillus johnsonii*; 1 × 10^9^ CFU/day	DSS (2%)	Male C57BL/6 mice; *n = 10;* 5/group	↑ Colon length ↓ Histology score ↑ Muc2 and ZO-1+ ↓ Macrophages infiltration ↑ F4/80+CD11b+Ly-6G ↑ IL-10 in ↑C206+ from BMDMs	↑ *Prevotellaceae,* ↑ *Clostridia,* ↑ *Bacteroidota*, ↓ *Actinobactriota*, ↓*Lachnospiraceae*, ↓*Erysipelotrichaceae*, ↓*Oscillospiraceae* ↓*Fermicuts*	-
[140]	Chinese fermented foods *Lactobacillus alimentarius* NKU556; 0.2 mg mL^–1^ Fe^2+^	DSS (4%)	Male BALB/C mice, *n = 60*; 12/group	↓ Colon shortening ↓ Histological score ↓ DAI ↓ Body weight loss ↓ TNF-α, IL-17, IL-1β, ↓ LCN2 ↑ Claudin-1, occluding and ZO-1 ↑ Hepcidin	-	↓MDA ↑SOD ↑GSH-PX
[141]	*Lactobacillus acidophilus*; 1×10^8^ CFU	DSS (5%)	Male Sprague Dawley rats; *n = 70;* 10/group	**Live *L. acidophilus*:** ↑ Body weight ↓ DAI ↑ Colon length ↓ Histologic score ↑ Occludin, Claudin, ZO-1 and TFF-3 ↓ TNF-α, IL-6 and MCP-1 ↑ IL-10 ↓ NLRP3, Caspase 1 and ASC ↓ IL-1β and IL-18 ↑ LC3II/I and ↓ P62	↑ Fecal acetic acid ↑ Propionic acid ↑ Butyric acid ↑ Caproic acid ↑ *Blautia fae* ↑ *Faecalibacteria prausnitzii* ↑ *Rumicococcus torques*	↑ GSH-PX ↓ MDA ↑ CAT
[142]	*Lactobacillus plantarum* CBT LP3; 1 × 10^8^ CFU/day	DSS (2.5%)	Female C57BL/6 mice; *n = 25;* 5/group	↓ Body weight loss ↓ DAI ↓ Histomorphological score ↑ Colon length ↑ Goblet cell count ↓ TNF-α, IL-1β and IL-17	-	↓ iNOS
[143]	*Lactobacillus salivarius;* 1 × 10^9^ CFU/day	DSS (2.5%)	C57BL/6JOlaHsd mice; *n = 16;* 4/group	↑ Blood score ↓ Stool score ↓ DAI ↑ Colon length ↑ Transepithelial resistance ↑ IL-10	↑ *Verrucomicrobia* ↑ *Lactobacillus* ↑ *Clostridia XIVa* ↑ *Akkermansia* ↓ *Prevotella* ↓ *Alloprevotella*, ↓ *Bacteroides* ↓ *Porphyromonadaceae* ↓ *Alistipes*	↓ MPO activity
[144]	*Lactobacillus acidophilus*@hyaluronic acid grafted with phenylboric acid (Lac@HDP); 1 × 10^9^ CFU	DSS (3%)	Female C57BL/6 mice: *n = 20*; 5/group	↑ Colon length ↓ IL-6, IL-1β, TGF-β ↑ IL-10 ↑ ZO-1 and occludin-1	↑ Bacterial counts ↑ Adhesion ability Lac@HDP ↑ Bacterial abundance ↓ *Desulfovibrionaceae* ↑ *Lactobacillus* ↑ *Akkermansia*	-
[146]	*Porphyromonas gingivalis* and *Lactobacillus rhamnosus* GG probiotics; 50 μg mL^−1^	DSS (3%)	Female C57BL/6 mice; *n = 25*; 5/group	↑ Colon length ↓ DAI ↓ Histological activity index ↓ IL-17/ Foxp3+ cell ratio ↓ Th17/ Treg ratio ↓ IL-17A, IL-17F and IL-6	-	-
[148]	*Lactobacillus plantarum strains;* 1 × 10^9^ (Low dose) or 1 × 10^10^ (High Dose) CFU/mL/day	DSS (3.5%)	BALB/c mice Sex n/i; *n = 60;*12/group	**Low Dose:** ↑ Body weight change% ↓ DAI ↑ Colon length ↑ IL-10 ↓ TNF-α, IL-1β and IL-12 **High Dose:** Same results as the ‘low dose’ PLUS ↓ Histological score ↓ TLR-4 ↓ MyD88, *p*-p65 and *p*-IkB	**High Dose:** ↑ *Butyricoccus* ↑ *Bacteroides* ↑ *Lachnospiraceae_NK4A136* ↑ *Lactobacillus* ↑ *Bifidobacterium* ↑ *Turicibacter* ↑ *Faecalibacterium* ↓ *Campylobacter* ↓ *Aliestipes* ↓ *Parabacteroides* ↓ *Alloprevotella* ↓ *Helicobacter* ↓ *Desulfovibrio* ↓ *Odoribacter* ↓ *Blautia* ↓ *Escherichia-Shigella*	**Low and High Dose:** ↓ MPO activity
[149]	*Lactobacillus casei* LH23; 1 × 10^8^ CFU/day	DSS (2.5%)	Female C57BL/6J mice; *n = 32;* 8/group	↑ Body weight change ↓ DAI ↑ Colon length ↓Colon pathology/inflammation score ↓ Cd68 ↓TNF-α, IL-1β and IL-6 ↑ Foxp3 ↑H3K9 acetylation	↑ Acetic acid ↑ Propionic acid ↑ Butyric acid	↓ MPO activity
[150]	*Lactobacillus brevis-*derived long-chain polyphosphate; 5 μg/mice/day	DSS (2.5%)	BALB/c Wistar Rattus: *n = 16*, 8/group	↓ Histopathological score ↓ Shortening of colon, ↓ IFN-γ, IL-6, TNF-α and IL-1β ↑ CD61	-	-
[151]	*Bifidobacterium bifidum*, *Enterococcus faecalis*, *Lactobacillus rhamnosus, Lactobacillus fermentum;* 1 × 10^9^ CFU	DSS (3%)	Male C57BL/6N mice; *n = 90;* 10/group	↓ Body weight loss, ↓ DAI ↑ Colon length ↓ TNF-α, IL-1β, IL-6 ↑ ZO-1, Claudin-4 and Occludin ↑ MUC2	-	-
[152]	*Bifidobacterium bifidum* BGN4-SK probiotic; 1 × 10^10^ CFU/day	DSS (2%)	Female C57BL/6 mice; *n = 48;* 8/group	↓ Body weight change ↓ Histopathological score ↓ TNF-α, IL-1β, IL-8 and IL-6 ↑ Claudin and ZO-1	-	↑ SOD ↑ GSH-Px ↑ CAT ↓ MPO
[153]	*Bifdobacterium animalis* spp. lactis (Bl 5764) and *Lactobacillus reuteri* (Lr 5454) probiotics; 1 × 10^8^ CFU/day	TNBS (110 mg/kg)	Female C57BL/6J and BALB/c ByJ mice; *n = 40;* 5–10/group	↓ Body weight loss ↓ Macroscopic score ↓ IL-1β and IL-6 ↓ Mip2 and tnf-α	-	-
[155]	*Pediococcus pentosaceus;* 1 × 10^9^ CFU /day	DSS (2%)	Male C57BL/6 mice; *n = 12*; 6/group	↑ Body weight and colon length ↓ Histopathology score ↓intranuclear TUNEL positive area ↑ ZO-1 and occludin ↑ Bcl2/Bax ↓ M1 and ↑M2 macrophages ↓ NF-κB p65 ↓ IL-1β and IL-6	↑ Diversity, ↑ *A. muciniphila* ↑ *C. cocleatum* ↑ *A. massiliensis*, ↑ *B. pseudolongum* ↑ *Verrucomicrobiaceae abundance.*	↓ H_2_O_2_
[156]	*Akkermansia muciniphila;* 1 x 10^9^ CFU/day.	DSS (3.0%) mice only	UC patients *n = 12;* Male C57BL/6 mice; *n = 10;* n = 3–6/group	**UC patients (untreated):** ↓*A. muciniphila* **Mice:** ↑ Colon weight % and length ↓ Histology score ↑ Goblet cells ↑ NLRP3 ↓MCP-1 and IL-6	-	-
[157]	*Akkermansia muciniphila* 139 and ATCC; 2 × 10^8^ CFU/ml/day	DSS (3%)	Male C57/BL6 mice; *n = 40;* 10/group	↑ Body weight change%, ↓ LCN2 ↓ Histological score ↓ IL-1β, IL-6, IFN-γ ↑ Foxp3+Cells and GPR43 ↑ Occludin, cldn3 and Muc2	↑ Relative abundance ↑ Acetate ↑ Propionate ↑ Butyrate	**-**
[158]	*Saccharomyces cerevisiae* 39#; 1 × 10^9^ CFU/mL	DSS (2%)	Male C57BL/6 mice; *n = 26*; 6/group	↓ DAI ↑ Colon length, ↓ Histological score ↑ MUC2 goblet cell/crypt ↑ ZO-1 staining ↓ TNF-α, IL-6, GSDMD, ↓ NLRP3, and IL-1βp17.	↑ *Lachnospiraceae*	↓MPO ↓NO

**BMDMs**, Bone marrow-derived macrophages; **CXCL-1**, Chemokine ligand-1; **CCL2**, Chemokine ligand 2; **CAT**, Catalase; **CFU**, Colony-forming unit; **Dact3**, Dishevelled Binding Antagonist Of Beta Catenin 3; **DSS**, Dextran sodium sulfate; **DAI**, Disease Activity Index; **DNBS**, Dinitrobenzenesulfonic acid; **EcN**, *Escherichia coli* Nissle; **FOXP3**, Forkhead box P3; **GSDMD**, Gasdermin D; **GPR43**, G-protein coupled receptor; **GSH-Px**, Glutathione peroxidase; **hu-FMT**, Human fecal microbiota transplantation; **H_2_O_2_**, Hydrogen peroxide; **IFN-*γ***, Interferon gamma; **iNOS**, inducible nitric oxide synthase; **NF**-**κB**, Nuclear factor kappa B; **n/i**, no-information provided; **LCN2**, lipocalin-2; **LC3II/I**, Isoform II/I of microtubule-associated protein 1 light chain 3; **LPF**, live *P. freudenreichii* KCTC 1063; **LPO**, Lipid peroxidase level; **mix-sup,** Supernatant mixture; **Mip2**, macrophage inflammatory protein 2; **MYD88**, Myeloid differentiation primary response 88; **MCP-1**, Monocyte chemoattractant protein-1; **MDA**, Malondialdehyde; **MPO**, Myeloperoxidase; **NLRP3**, NOD-like receptor protein 3; ***p*-IkB**, Phospho- IkappaB kinase; **SPFC**, Supernatant of the *P. freudenreichii* culture; **SOD**, Superoxide dismutase; **TNBS**, Trinitrobenzenesulfonic acid; **TGF-β**, Transforming growth factor- beta; **TNF-α**, Tumor Necrosis Factor-alpha; **TFF-3**, Trefoil factor 3; **Treg**, T regulatory; **TLR4**, Toll-like receptors-4; **ZO-1**, zonula occludens 1.

### Probiotics as an anti-inflammatory adjuvant therapy

Microbiota have been considered a key factor in the development of IBD, which can be modified by diet and/or supplements using beneficial bacteria or beneficial compound derivates from bacteria (post-biotic) [137,138]. Of note, *Lactobacillus* and *Bifidobacterium* strains are more likely to be studied in the management of IBD. For instance, *Lactobacillus* administration led to significant amelioration of disease severity including reducing goblet cell and colonic crypt damage, improvement in DAI, colon shortening, inflammatory cell infiltration, epithelial damage, inflammatory factor expression, and migration of CD206^+^ macrophages into the colon tissue [139–143]. Furthermore, the expression of proteins such as occludin, claudin-1, and ZO-1 was significantly elevated with *Lactobacillus* supplementation [140,144], leading to enhanced intestinal permeability and barrier function [145]. Significantly lowered Th17 cell level and elevated Treg cells were also observed in parallel, implying probiotics’ ability to regulate T cells [146]. The evidence for specific bacterial strain and their impact on IBD is listed below:

- *Lactobacillus rhamnosus* strain GG has not shown robust evidence to induce or maintain remission in CD after 6 months [147]. Such difference in the therapeutic effect of *Lactobacillus* may be dependent on the presence of ongoing insult or damage. *Lactobacillus rhamnosus* GG showed greater anti-apoptotic effect when combined with a pathogen bacteria [146], implying probiotics’ effect may depend on threat or damage.
- Live *Lactobacillus acidophilus* significantly inhibited IL-18, IL-1β, NLRP3 activation, enhancing autophagy. However, no reduction of these inflammatory markers was observed when *L. acidophilus* was provided in non-viable bacterial or supernatant (containing secreted compound) forms [141].
- *Lactobacillus salivarius* UCC118 improved IL-10 level including M2 macrophages but showed no significant difference in terms of colon shortening, and intestinal permeability shown by tight junction protein expression [143].
- *Lactobacillus plantarum* strains showed efficacious effects on body weight, colon length, and anti-inflammatory cytokine production. In addition, high dose of *L. plantarum* L1 markedly diminished DAI score and simultaneously reduced pro-inflammatory cytokine production, by downregulating the expression of TLR4, MyD88, and NF-κB [148].
- *Lactobacillus casei* strains LH23, LH1129, and LH1134 showed significantly elevated capacity to adhere to mucosal cells in *in vitro* studies, indicating its potential to be more effective compared to other strains with lower adhesive qualities. Improved SCFA level and promotion of Treg differentiation lead to diminished immune responses [149].
- *Lactobacillus brevis*-derived long-chain polyphosphate significantly increased platelet accumulation and aggregation and *in vitro*, leading to enhanced healing of the colonic mucosa. Interestingly, it did not significantly elevate other known factors contributing to mucosal healing such as vascular endothelial growth factor (VEGF), epidermal growth factor (EGF), platelet-derived growth factor (PDGF), and transforming growth factor-β (TGF-β) [150], suggesting its mechanism of action remains to be identified.

On the other hand, the most frequently investigated probiotic as an alternative treatment for IBD is *Bifidobacterium.* Notably:

- *Bifidobacterium bifidum* strains (FL-276.1 and FL-228.1) are generally considered to offer the best beneficial impact on IBD when compared with other strains or the anti-inflammatory agent 5-ASA. For example, the administration of FL276.1 and FL228.1 ameliorates the decline in DAI and body weight while also inhibiting colon shortening. Additionally, mice treated with these two strains showed an up-regulation of the ZO-1, claudin-4, occludin, and Muc2 genes in colon tissue, while TNF-α, IL-1β, and IL-6 were downregulated [151].
- *Bifidobacterium bifidum* BGN4 significantly ameliorated the symptoms of DSS-induced colitis, increasing the expression of tight junction genes and decreasing pro-inflammatory cytokines such as IL-6, IL-1β, and TNF-α [152].
- *Bifdobacterium animalis* spp. Lactis (BI 5764) strain showed antimicrobial effect against *C. rodentium* infection, which was used as an equivalent for human *E. Coli* infection [153].

The lactic acid-producing probiotic *Saccharomyces cerevisiae* (*S. cerevisiae*), *Akkermansia muciniphila*, lactic acid bacteria *Pediococcus pentosaceus* (*P. pentosaceus*), *Clostridium butyricum*, and *E. coli* Nissle 1917 have all demonstrated some anti-inflammatory action as judged by decreasing inflammatory cytokine levels, goblet cell loss, mucosal damage, body weight loss among others, which can be seen in Table 8 [35,154–158].

### Probiotics as an anti-oxidative adjuvant therapy

In most cases, studies with supplemented probiotics showed an overall antioxidant activity [35,140,142,152,154,158]. For instance, the administration of 2 × 10^8^ CFU mL^−1^ at 0.5 mL day^−1^ of *L. acidophilus* for 8 days, regardless of whether it is administered live, heat killed or as a supernatant form, consistently showed a significant elevation of serum glutathione-PX (GSH-PX) which contributes to total plasma antioxidant capacity. However, a reduction of lipid oxidation (judged by comparing levels of MDA) was only observed with the use of live *L. acidophilus* [141]. Similarly, the administration of 1 × 10^10^ CFU of *Bifidobacterium bifidum* BGN4-SK for 8 days led to significant elevation of serum GSH-PX, and both CAT and SOD antioxidant enzymes [152]. Furthermore, *S. cerevisiae*, *P. pentosaceus, Lactobacillus plantarum*, and *alimentarius*, liposome-coated *E. coli* Nissle 1917 (Pt-Lipid@EcN) and *Clostridium butyricum*-derived extracellular vesicles have demonstrated significant antioxidative effect through the repression of MDA, inducible NO synthase, and MPO activity, which relates to diminished total ROS production [35,140,142,152,155,158]. More information can be found in Table 8.

However, differences in the effects, and whether there is improvement in the markers of IBD, are largely dependent on the strain being supplemented. For instance, *B. bifidum* strain BGN4 and BNG4-pBESIL10 significantly diminished CAT, GSH-Px, while in contrast *B. bifidum* strain BGN4-SK led to significantly higher CAT, GSH-Px, and SOD, indicating protection against oxidative stress [152]. Although *Lactobacillus salivarius* UCC118 did not significantly reduce MPO levels [143], both *Lactobacillus casei* (LH23) and *Lactobacillus plantarum* (L15) significantly suppress MPO activity, indicating suppressed neutrophil recruitment or activation [148,149]. Even within *Lactobacillus acidophilus* as a supplement, significant differences in the capacity to influence lipid oxidation (MDA) or impact high-molecular weight antioxidant levels (CAT) are obtained depending on whether it is delivered as supernatant, heat killed, or live forms [141].

### Probiotics as modulators of the gut microbiota

Dysbiosis in IBD is characterized for an alteration in maintaining gut homeostasis and SCFAs producing bacteria, especially loss in the production of butyrate and propionate [159]. In contrast, sulfate-reducing bacteria (SRB) are suggested to be involved in the process of bowel inflammation due to the production of hydrogen sulfide (H_2_S) that is also a redox active gas. There is evidence that *Desulfovibrio vulgaris*, the most predominant SRB, may induce intestinal permeability through the involvement of H_2_S. Thus it may be inferred that microbiota may be a factor of influence for IBD pathophysiology, and supplementing probiotics may be beneficial to alleviate IBD severity through secondary effects following adjustment of the individual’s microbiome state as mentioned before [160].

Although there may be difference between the species and the strains, probiotic microbiota showed certain overlap in its effect on microbial composition and abundance. The administration of *S. cerevisiae* and lactic acid significantly improved fecal microbial abundance and diversity [158]. Overall, *Lactobacillus* genus led to significant reduction in number of pathogenic microbiota while improving α-diversity and abundance [143,144]. The administration of 1 × 10^9^ CFU/ml *Akkermansia muciniphila* strains led to significant amelioration of gut microbiota diversity [154]. Overall, probiotic species led to increased diversity of microbiota.

In general, elevation of the abundance of *Bacteroides*, *Lactobacillus*, *A. muciniphila*, *Akkermansia*, *Prevotellaceae*, *Akkermansia*, and *Verrucomicribiaceae* were seen in animal models when they were supplemented with probiotic therapy [139,143,154,155]. The suppression of pathogenic factors was seen in both *Lactobacillus* strains and *Clostridium butyricum*-derived extracellular vesicles [148]. A similar outcome was obtained with *Lachnospiraceae*, where its elevation was accompanied by significant increase in fecal SCFA [158]. Thus probiotic’s effect on microbiota may be intricately dependent on the fine-tuning combinations of different strains or species to yield synergistic or conflicting effects.

## Discussion

This comprehensive review was conducted to provide, for first time, an evaluation of the most recent literature evidence on a role for natural products as potential therapeutics for the treatment of IBD. While studies have demonstrated the efficacy of individual natural products and their anti-inflammatory, gut microbial, and anti-oxidative actions, there remains a significant knowledge gap in the comparison between these individual natural products and whether combinations can provide synergistic or additive benefits beyond their individual activities. Furthermore, the evidence indicates a holistic perspective in the use of CAM and the complexity of its compounds, which can enhance various biological states, act through multiple mechanisms, and potentially reduce the required dosage of conventional drugs, thereby minimizing side effects. This contrasts with the classical approach of single-targeted therapeutics. Also, this review may serve as a broad guide to assist in developing informed decisions about using natural ingredients with intended therapeutically beneficial outcomes. On balance, most of the studies included in this review support the efficacy of natural products with bioactivities compounds such as polyphenols, polysaccharides, emodins, SCFA, and probiotics being prominent in the recent body of literature. These compounds have for the most part all shown high efficacy for anti-inflammatory action, restoration of homeostatic gut microbiome, and enhanced antioxidant activity in the context of IBD.

Studies identifying the biological impact of polyphenols generally showed consistent macroscopic attenuation of inflammation and suppression of biomarkers, while promoting M2 macrophage phenotype, enhancing butyrate-producing microbiota abundance and anti-oxidative biomarkers [26,28,39,40,51]. Data compiled from studies focused on polysaccharides showed similar attenuation of macroscopic signs and inflammatory biomarkers [58,59,60,61,62], however, the evidence was not fully clear and some studies were inconsistent, alluding to the requirement of standardized individual dosing, which may also vary for specific types of polysaccharides [63,64,66]. Interestingly, polyphenols and polysaccharides both enhanced SCFA productions via the modulation of gut microbiota [40,58] and both SCFA-enhancing natural product and SCFA itself exhibited a significant effect on promoting anti-inflammatory M2-like macrophages, which has been linked to be involved in the IBD pathophysiology [161]. Thus further study may be needed to determine whether polyphenol induction of altered SCFA production or the direct supplementation of SCFAs via over-the-counter products may be more consistent in providing benefit to subjects diagnosed with IBD.

Similar to polyphenols and polysaccharides, the anthraquinone emodin showed anti-inflammatory effect, anti-oxidation properties, and restoration of gut microbiota [80,81,82]. However, there are only few studies confirming the therapeutic effect of this compound as a treatment for IBD. Therefore, there is insufficient available evidence to draw any solid conclusions about the management of IBD with this class of compound.

Notably, emerging studies reported the role of SCFA in IBD management, which has shown a direct association with beneficial gut microbiota, mucin production, intestinal barrier, and mucus homeostasis [162]. First, butyrate showed damage-dependent anti-inflammatory effect and a significant association with gut microbiota [12,102,108], especially butyrate leading to significantly increased *Firmicutes* and *Lactobcillaceae*, although its dependency on microbiota was also questioned by a study involving antibiotic therapy [109]. Second, propionate displayed positive association with microbiota producing anti-inflammatory activity [111], although some investigations yielded non-significant results [112]. Additionally, it has the potential to support pathogenic microbiota by enhancing biofilm formation [113]. Acetate, like polysaccharides, exhibited anti-inflammatory effects [106,116–119,163] but its bioactivity was only partially microbiota-dependent in certain studies [116,120]. It showed significant interrelationship with gut microbiota [117] and its effect may be influenced by an individual’s ability to produce and respond to SCFA [122]. In contrast, the inhibition of butyrate’s anti-inflammatory action showed a link to UC, clearly identifying a connection between reduced SCFA action and diseased state. Thus although there is contrasting results in the available literature there is increasing evidence to indicate that SCFA production has an impact of altering gut microbiota while also acting through a mechanism of anti-inflammation, likely through the gut microbiota–inflammation axis.

Finally, probiotics supplementation has showed anti-inflammatory and antioxidant effects, as well as the ability to modulate the gut microbiome. Gut microbiota not only affect the large intestine, but may also have systemic effect through its release of metabolites including SCFAs, amino acids, and dopamine, GABA, acetylcholine, and noradrenaline [164]. The association between gut microbiota and inflammatory state has been shown as predictors of several disease [165,166]. This has been supported for IBD as well, with IBD patients showing significantly lower abundance and composition compared to population cohort or healthy controls [167,168]. In this review, the collected studies showed that probiotics effectively ameliorated inflammation and gut dysbiosis, but differences in correlation with *Firmicutes* species were observed (refer to Table 8).

**Figure 3.**
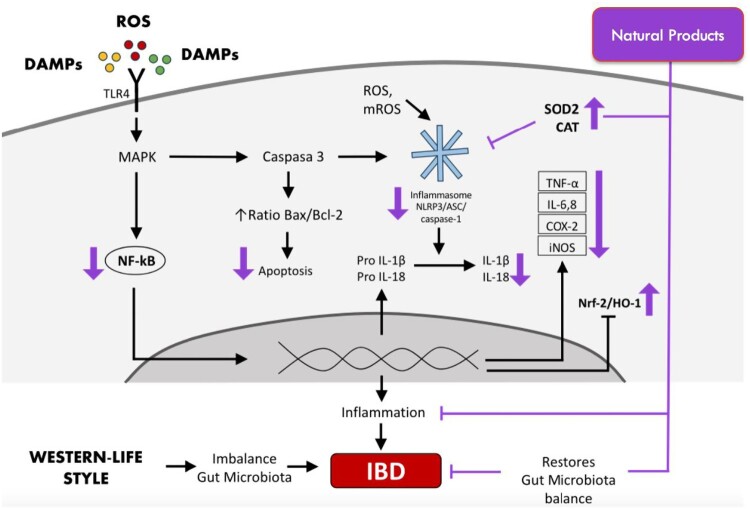
Schematic diagram of effect of natural products on various aspects of IBD pathophysiology. Reactive Oxygen Species (ROS) and Damage Associated Molecular Patterns (DAMPs) bind to pattern recognition receptors such as Toll-Like Receptor 4 (TLR4) to induce the activation of Mitogen Activated Protein Kinase (MAPK)/NF-kB pathway, as well as the activation of apoptosis through caspase 3 activation. Natural products have shown the alleviation of IBD in different points of pathogenesis: inhibition of NF-kB and apoptosis, suppression of inflammasome and pro-inflammatory cytokines, and an increase in antioxidant enzymes (SOD2, CAT, Nrf-2/HO-1). Combined with this, by restoration of gut microbial balance, natural products may impact on different aspects of IBD pathophysiology leading to the suppression of IBD.

In summary, the gathered findings support the role of gut microbiota’s modulation in IBD therapy. On the another hand, high fiber diet and high fermented food altered microbiome function and diversity respectively, positively influencing the immune response. Indeed, fermented foods showed steadily increased microbiota diversity and decreasing inflammatory markers in healthy individuals [169]. Similarly, diet consisting of high fructose significantly altered gut microbiota and elevated pro-inflammatory cytokine infiltration in the colon, while small amount did not show significant inflammatory changes [170]. This alludes the potential of gut microbiota alteration via diet and its impact in inflammatory pathway. However, as there are far more *in vitro* studies compared to *in vivo*, this review collected *in vivo* studies and showed significant gut microbiota restoring effect of natural products as mentioned above. Regarding the antioxidant effect, the suppression of MDA (oxidative damage), and elevation of SOD and GSH (both antioxidants) were the most consistent outcomes observed in natural products. Polyphenols, polysaccharides, emodin, butyrate, propionate, acetate, and probiotics depending on the strain (refer to Table 1), all successfully displayed the capacity to modulate oxidative stress status. This antioxidant property is also link to inflammatory response. These total effects can be seen in Figure 3, on how natural products may lead to the suppression of IBD pathophysiology.

In conclusion, when comparing the effects between natural products, polyphenols displayed the most consistent results in its anti-inflammation, gut microbiota restoration, and anti-oxidation. A well-informed understanding of the potential therapeutic role of CAM – specifically polyphenols – can be used as a guide for patients in the treatment of IBD and its associated symptoms, specifically as they increasingly turn to alternative therapies as complementary options to conventional treatments.

## Limitations and future direction

Most of the studies included in the review are preclinical *in vivo* animal studies, and the efficacy of natural products may differ when translated to human studies. Additionally, most of the included studies compare between healthy mice, IBD-induced mice via TNBS, DNBS, or DSS, with natural product therapy, which simulated the physiopathology of the disease but not the etiopathogenesis, making it difficult to achieve a complete understanding of potential therapeutic and to reach a comprehensive approach of natural products as a treatment for IBD patients. Moreover, many studies often compare the efficacy of natural products versus conventional IBD medications, but not combination of conventional IBD medication and natural products. Thus its results are more representative for when using the natural products alone and not using it as a supplementary to conventional IBD medication. This study does not take account the interaction which may occur when combining natural products and conventional IBD medication together, which may alter its efficacy and safety. Efficacy may vary by individuals due to metabolism, and advising a single dose for everyone may not work. Individuals may have to trial different natural products themselves to end up with optimal result.

In a nutshell, more clinical studies are necessary to compare the bioactivity of natural products versus conventional therapy to propose them as an effective and safe alternative as a complementary treatment for patients diagnosed with IBD. However, the accumulated evidence from preclinical (animal) models would support the need for such large-scale clinical trials.

## Data Availability

No datasets were created or analyzed during the course of the current study. Therefore, data sharing is not applicable.

## Abbreviations

**Abbreviations:** 4- HNE, 4-hydroxynonenal; 5-ASA, 5-aminosalicylates; ABX, antibiotic; ACAT1, acetyl-CoA acetyltransferase 1; AIEC, a strain of adherent-invasive *E. coli*; AKT, protein kinase B; AMP, atractylodes macrocephala Koidz polysaccharide; AOM, azoxymethane; AOS, alginate oligosaccharides; APS3a, non-honey-processed Astragalus polysaccharides; Arg-1, arginase 1; ASC, apoptosis-associated speck-like protein containing a caspase recruitment domain; ATB, antibiotic properties; BCFA, branched-chain fatty acids; BDH1, 3-hydroxybutyrate dehydrogenase I; BHB, B-hydroxybutyrate; BI, bilobalide; BMDMs, bone marrow-derived macrophages; CA, caffeic acid; CACC, colitis-associated colorectal cancer; CAM, complementary and alternative medicine; cAMP, cyclic AMP; CAT, catalase; CC, colorectal cancer; CCL2, chemokine ligand 2; CD, Crohn’s disease; CFU, colony-forming units; CGA, chlorogenic acid; Chil3, chiinase-like protein; CitH3, citrullinated H3; CLP, cecal ligation and puncture; COVID, coronavirus disease-2019; COX-2, cyclooxygenase-2; CP, calprotectin; CRP, C-reactive protein; CXCL-1, chemokine ligand 1; CXCR2, interleukin 8 receptor; Cy, cyclophosphamide; Dact3, dishevelled binding antagonist of beta catenin 3; DAI, disease activity index; DAMPs, damage associated molecular patterns; DAO, diamine oxidase; DAPP, dried apple peel powder; DCA, dichloroacetate; DCs, dendritic cells; DNBS, dinitrobenzene sulfonic acid; DO, *Dendrobium officinaleon*; DSS, dextran sodium sulfate; *E. coli*, *Escherichia coli*; EA, ellagic acid; EC, *Eucheuma cottonii*; EcN, *Escherichia coli* Nissle; EGCG, epigallocatechin-3-gallate; EGF, epidermal growth factor; EMO/PSM NPs, emodin-loaded poly (DL-lactide-co-glycolide)/Eudragit S100/montmorillonite nanoparticles; Emodin, 1,3,8-trihidroxy-6-methyl-anthraquinone; EPS1-1, *Rhizopus nigricans* extracellular polysaccharide; ER, endoplasmic reticulum; ERK, extracellular signal-regulated kinase; F/B, *Firmicute* to *Bacteroidete*; F12, probiotic microparticles; FA, ferulic acid; FCP, fecal calprotectin; FFAR, free fatty acid receptors; FMT, fecal microbiota transplant; FOXO3, forkhead box O3; GM-CSF, granulocyte-macrophage colony-stimulating factor; GPCRs, G protein-coupled receptors; GSDMD, Gasdermin D; GSH-PX, glutathione-PX; GSH, glutathione; GSP, grape seed proanthocyanidin; GST, glutathione-S-transferase; H_2_O_2_, hydrogen peroxide; H_2_S, hydrogen sulfide; HAPS3a, honey-processed Astragalus polysaccharides; HAW1, *Crataegis pinnatifida* (Hawthorn); HC, healthy volunteers; HDAC, histone deacetylase; HE, *Hericium erinaceus*; HMGB1, high mobility group protein B; HMGCS2, 3-hydroxymethylglutaryl-CoA synthase 2; HO-1, heme oxygenase-1; hu-FMT, human fecal microbiota transplantation; i.v, intra-vascular; IBD, inflammatory bowel disease; IC, indeterminate colitis; IFN, interferon; IKK, inhibitory Kappa B kinase α; IL, interleukin; INCLD, international cohort on lifestyle determinants; iNOS, inducible nitric oxide synthase; JAK2, janus kinase 2; JNK, c-Jun N-terminal kinase; LA-GOS, *Lupinus albus* α-galacto-oligosaccharides; Lac@HDP, *Lactobacillus acdiphilus*@Hyaluronic acid grafted with dopaine protected by phenylboric acid; LC3, microtubule-associated protein 1A/1B-light chain 3; LC3II/I, isoform II/I of microtubule-associated protein 1 light chain 3; LCN2, lipocalin-2; LPF, live *P. freudenreichii* KCTC 1063; LPO, lipid peroxidase level; MAPK, mitogen activated protein kinase; MCP-1, monocyte chemoattractant protein-1; MDA, malondialdehyde; Mip2, macrophage inflammatory protein 2; mix-sup, supernatant mixture; MPO, myeloperoxidase; Mt., mitochondria; MUC, mucin; MYD88, myeloid differentiation primary response 88; n/i, no information provided; NaB, sodium butyrate; NE, neutrophil elastase; NET, neutrophil extracellular trap; NF-κB, nuclear factor kappa light chain enhancer of activated B cells; NFATC, nuclear factor of activated T cells; NK, natural killer; NKU556-Fe, *Lactobacillus alimentarius* NKU556 iron-enriching from Chinese fermented food; NLRP3, nucleotide-binding domain, leucine-rich containing family, pyrin domain containing 3; NO, nitric oxide; NO_2_, nitrite; NO_3_, nitrate; Noni-PLS, isolated polysaccharide from *Morinda citrifolia* Linn; NPs, nanoparticles; NQO1, NADH-quinone reductase; Nrf-2, nuclear factor erythroid 2-related factor 2; Olfr78, olfactory receptor 78; ORY, food-derived oryzanol; OTUs, operational taxonomic units; p-IkB, phospho-I kappa B kinase; PCC, protein carbonyl compounds; PCNA, proliferating cell nuclear antigen; PDGF, platelet-derived growth factor; Poly P, long-chain polyphosphate; PPM, parts per million; PRISMA, preferred reporting items for systematic review and meta-analysis; PRR, pattern recognition receptors; Pt-Lipid@EcN, liposome-coated *Escherichia coli* Nissle (EcN) 1917; PYY, peptide YY; Redox, reduction–oxidation; RGal, Rhamnogalacturonan; RNS, reactive nitrogen species; ROS, reactive oxygen species; SCFA, short-chain fatty acids; SDEA, *Selaginella doederleinii* Heiron etyl acetate; SFE, *Sophora flavescens* extract; sIgA, secretory immunoglobulin A; SIRT1, Sirtuin 1; SOD, superoxide dismutase; SPDEF, SAM pointed domain containing ETS transcription factor; SPFC, supernatant of the *P. freudenreichii* culture; SRB, sulfate-reducing bacteria; STAT3, signal transducer and activator of transcription 3; T-AOC, total antioxidant capacity; TED, trans-emodin dianthrones; TFF-3, trefoil factor 3; TFPS, *Camellia sinensis* L. pectic heteropolysaccharides; TGF-β*:* transforming growth factor-β; TLR: toll-like receptors; TNBS: trinitrobenzene sulfonic acid; TNF-α: tumor necrosis factor; Treg; T regulatory; UC: ulcerative colitis; UCDAI: UC disease activity index; UroA: Urolithin A; VEGF: vascular endothelial growth factor; W: week; ZAVE: Zhenjiang aromatic vinegar extract; ZO-1: zonula occludens 1
